# Spider‐Inspired Electrohydraulic Actuators for Fast, Soft‐Actuated Joints

**DOI:** 10.1002/advs.202100916

**Published:** 2021-05-29

**Authors:** Nicholas Kellaris, Philipp Rothemund, Yi Zeng, Shane K. Mitchell, Garrett M. Smith, Kaushik Jayaram, Christoph Keplinger

**Affiliations:** ^1^ Paul M. Rady Department of Mechanical Engineering University of Colorado Boulder CO 80309 USA; ^2^ Materials Science and Engineering Program University of Colorado Boulder CO 80303 USA; ^3^ Robotic Materials Department Max Planck Institute for Intelligent Systems Stuttgart 70569 Germany

**Keywords:** actuator, articulation, bioinspired, electrohydraulic, HASEL, SES, soft

## Abstract

The impressive locomotion and manipulation capabilities of spiders have led to a host of bioinspired robotic designs aiming to reproduce their functionalities; however, current actuation mechanisms are deficient in either speed, force output, displacement, or efficiency. Here—using inspiration from the hydraulic mechanism used in spider legs—soft‐actuated joints are developed that use electrostatic forces to locally pressurize a hydraulic fluid, and cause flexion of a segmented structure. The result is a lightweight, low‐profile articulating mechanism capable of fast operation, high forces, and large displacement; these devices are termed spider‐inspired electrohydraulic soft‐actuated (SES) joints. SES joints with rotation angles up to 70°, blocked torques up to 70 mN m, and specific torques up to 21 N m kg^−1^ are demonstrated. SES joints demonstrate high speed operation, with measured roll‐off frequencies up to 24 Hz and specific power as high as 230 W kg^−1^—similar to human muscle. The versatility of these devices is illustrated by combining SES joints to create a bidirectional joint, an artificial limb with independently addressable joints, and a compliant gripper. The lightweight, low‐profile design, and high performance of these devices, makes them well‐suited toward the development of articulating robotic systems that can rapidly maneuver.

## Graphical abstract

Spider‐inspired electrohydraulic soft‐actuated (SES) joints provide compliant, electrohydraulic actuation for articulated robots. These joints exhibit fast actuation, high forces, and large displacements. This article characterizes the quasistatic and dynamic performance of SES joints experimentally and presents a model of their quasistatic behavior. The versatility of SES joints is demonstrated in a bidirectional joint, an artificial limb, and a compliant gripper.

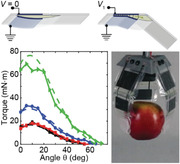

## 1 Introduction

The field of soft robotics endeavors to reproduce the versatility of natural organisms—in particular their ability to interact effectively with uncertain and dynamic external forces or environments—through the incorporation of elasticity and compliance into robotic structures.^[^ ^1^, ^2^, ^3^, ^4^, ^5^, ^6^, ^7^, ^8^, ^9^, ^10^, ^11^, ^12^ ^]^ Naturally, soft‐bodied animals^[^ ^13^ ^]^ that undergo continuum deformation, such as annelids,^[^ ^14^ ^]^ insect larvae,^[^ ^15^ ^]^ and molluscs,^[^ ^16^ ^]^ are often used as model organisms in soft robotics.^[^ ^17^, ^18^, ^19^ ^]^ However, the functional capabilities of these soft robots, such as weight support against gravity,^[^ ^20^ ^]^ body/appendage control,^[^ ^21^ ^]^ and rapid propulsion,^[^ ^22^ ^]^ could be further enhanced by incorporating arthropod‐inspired articulated exoskeletal mechanisms^[^ ^23^ ^]^ comprised of both rigid and compliant elements, all while maintaining impressive compliance, e.g., for navigating confined spaces.^[^ ^23^ ^]^

Amongst the arthropods, spiders (class Arachnida) feature one of the most successful and unique solutions for achieving motion in nature, as they integrate compliant articulation with fluidic actuation.^[^ ^24^ ^]^ Unlike most animals that use antagonistic muscle pairs for generating movement, spiders create leg extension through the use of hydraulic mechanisms, while using elastic elements or muscles for flexion.^[^ ^25^ ^]^ This hydraulic mechanism supplies the precise and coordinated motion needed to weave complex webs,^[^ ^26^ ^]^ as well as the powerful and fast maneuvers needed to hunt prey,^[^ ^27^, ^28^ ^]^ making spiders a prime source of bioinspiration for mechanisms of soft actuation.

Developing capable articulating robots that can reproduce animal‐like functionality necessitates the use of a lightweight actuation mechanism capable of high speed and high force output, large displacements, and compatibility with untethered operation. While myriad actuation strategies exist, no single technology yet satisfies all these requirements. Pneumatics—the most popular approach^[^ ^29^, ^30^, ^31^, ^32^ ^]^—requires bulky peripheral components such as tubes and valves and is plagued by tradeoffs between portability and speed: fast operation requires tethers to large reservoirs of pressurized fluid or pumps, while the speed of untethered systems is low.^[^ ^3^, ^17^ ^]^ Thermally driven systems achieve high specific energies, but demonstrate low bandwidth and efficiencies.^[^ ^33^, ^34^ ^]^ Piezoelectric mechanisms on the other hand exhibit high speed and portability,^[^ ^35^, ^36^ ^]^ but have limited displacement. Finally, dielectric elastomer actuators (DEAs) offer high efficiencies and speed,^[^ ^37^, ^38^ ^]^ even in untethered operation,^[^ ^39^, ^40^ ^]^ but require highly elastic dielectrics and stretchable electrodes^[^ ^41^ ^]^—both challenging material systems to work with—and the demonstrated torque production in articulated designs based on DEAs is low.^[^ ^42^, ^43^ ^]^

Electrohydraulic actuation mechanisms, as used in hydraulically amplified self‐healing electrostatic (HASEL) actuators, address some of these technological gaps by demonstrating many of the advantages of both fluidic and electrostatic actuators, with muscle‐like forces, high bandwidth, and promising efficiencies.^[^ ^44^, ^45^, ^46^, ^47^ ^]^ Peano‐HASEL actuators in particular exhibit attractive characteristics for bioinspired robotic systems due to their controllable linear contraction, scalable fabrication, and versatility in materials; however, the integration of Peano‐HASEL actuators into distributed articulating systems is difficult due to the need to convert linear movement into angular output; this requirement increases design complexity, introduces additional components, and limits the angular excursion of articulating structures.

Several pneumatic technologies have emphasized the advantages of mechanical anisotropy in articulating structures, including the integration of rigid structural components with soft actuation for articulation from mm to cm scales.^[^ ^30^, ^31^, ^48^, ^49^, ^50^ ^]^ In particular, the pouch motors introduced by Niiyama et al. provide a promising approach for articulation through their distributed design and control, but still suffer from the common pitfalls of pneumatics with the need for a supply of compressed air that is distributed through complex networks of lossy air lines; thus, these are still limited in their bandwidth and controllability for multiple degrees of freedom.

Here, to address the need for high‐performance actuators that enable fast articulation and can be seamlessly integrated into robotic structures, we introduce an actuation approach that enables a family of devices termed spider‐inspired electrohydraulic soft‐actuated (SES) joints. SES joints leverage qualities of both linear Peano‐HASEL actuators^[^ ^45^ ^]^ and rotational pouch motors^[^ ^31^, ^51^ ^]^ and feature high specific torque (comparable to electromagnetic servo motors), fast operation (demonstrated up to 24 Hz), low power consumption, and backdrivability, all in a low profile and lightweight design that exploits versatile fabrication methods. These SES joints leverage both rigid and compliant structural elements to produce a bioinspired high‐performance articulating mechanism that is driven by electrohydraulic principles. A quasi‐static model of SES joint performance is developed and experimentally validated in order to provide a tool for informing design improvements. To illustrate potential applications of these joints in robotics systems, we implement a range of structures with different functionalities such as a jumping robot that can leap over 10 times its height, a fast‐acting bidirectional joint that operates over 10 Hz, a multisegmented artificial limb with three independently addressable joints, and a three‐finger gripper capable of both delicate and powerful grasps. The structural simplicity of SES joints allows direct integration of actuation at the joint, with minimal peripheral components, thereby opening new opportunities for the creation of more complex, bioinspired structures with multiple degrees‐of‐freedom.

## 2 Results

### 2.1 Principles and Capabilities of SES Joints

#### 2.1.1 Principles of Operation for Spider Joints

**Figure 1:**
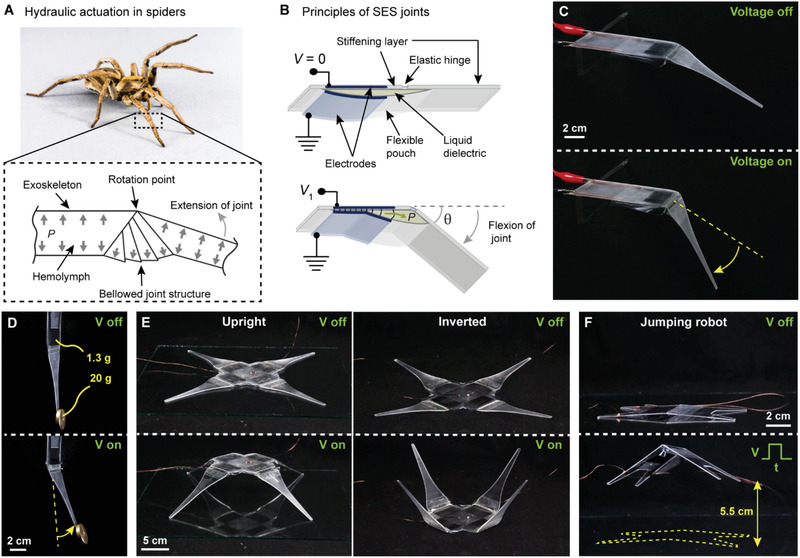
Principles of spider‐inspired electrohydraulic soft‐actuated (SES) joints. A) Hydraulic operation of the tibia‐metatarsus joint of a spider. Pressurization of hemolymph fluid causes the leg to extend. Adapted with permission.^[^ ^51^ ^]^ Copyright 2014, IEEE. A wolf spider (family Lycosidae) is pictured. B) SES joints consist of a flexible pouch filled with liquid dielectric, and a pair of opposing electrodes on the outside. A stiffening layer is placed on one side to constrain actuation while a flexible hinge provides stability and a passive restoring force. On application of voltage, Maxwell stress causes the electrodes to zip together progressively, which pressurizes the liquid dielectric (*P*) and causes flexion of the joint to angle *θ*. C) SES joint with voltage off and with voltage on (8 kV applied), which causes flexion of the joint. D) The rigid stiffening layer supports efficient force transfer along the limb; here, a 1.3 g actuator is lifting 20 g almost 10 cm away from the point of rotation. E) Multiple SES joints can be combined to create different types of robotic structures. F) SES joints feature excellent power‐to‐weight ratio and can be used to create jumping robots.

The basic structure of a spider leg joint^[^ ^25^, ^52^ ^]^ creates extension through hydraulic pressure (**Figure** **1**A). A stiff exoskeleton is segmented by a compliant joint with a soft bellowed membrane on the ventral side of the joint. The lacuna—the empty space in the exoskeleton—contains the spider's hemolymph, which acts as both its “blood” and a hydraulic fluid.^[^ ^53^ ^]^ Hemolymph is pressurized by muscles in the prosoma of the spider, and the soft bellowed membranes in the joint expand on pressurization, causing the leg to extend.^[^ ^29^, ^50^ ^]^ Restoring forces to provide flexion rely on either passive elastic components or muscles within the exoskeleton.^[^ ^54^ ^]^

#### 2.1.2 Principles of Operation for SES Joints Analogous to Spiders

Our SES joints aim to combine many of the central characteristics of the anatomy of spider joints to recreate their functionalities (Figure 1B). They use an electrohydraulic driving component that consists of 1) a flexible pouch made from dielectric film filled with 2) a liquid dielectric, and 3) a pair of flexible electrodes placed on opposing sides of the pouch (Figure 1B). This electrohydraulic component is integrated with a passive stiffening layer that acts as the exoskeleton to provide support and selective deformation by constraining one side of the liquid‐filled structure; an elastic hinge is located along the stiffening layer, which allows flexion of the joint, and provides an elastic restoring force, similar to that used in many spiders.^[^ ^25^, ^54^ ^]^ On application of DC high voltage (on the order of kilovolts), Maxwell stress causes the electrodes to zip together progressively, pressurizing and pumping the fluid;^[^ ^45^, ^46^, ^47^, ^55^ ^]^ the hydrostatic pressure coupled with the selective constraints causes flexion of the joint (Figure 1B,C; Movie S1, Supporting Information). The unique combination of electrostatic and hydraulic forces coupled with discrete stiffening layers results in a soft‐actuating joint with capabilities that are attractive for robotics applications such as efficient force transmission (Figure 1D), backdrivability (Movie S2, Supporting Information), the ability to be parallelized (Figure 1E; Movie S3, Supporting Information), and fast and strong actuation that enables the creation of robots that can leap into the air over ten times their body height (Figure 1F; Movie S4, Supporting Information).

#### 2.1.3 Fabrication Procedure for SES Joints

**Figure 2:**
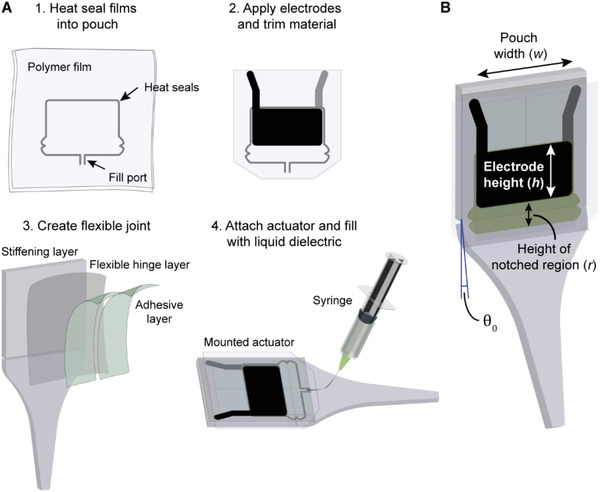
Fabrication process for SES joints. A) Representative fabrication steps for the material systems used in this paper. 1) Two layers of polymer film are heat sealed together to define a pouch shape, leaving a small opening at the bottom for filling. 2) Carbon electrodes are screen printed on either side of the pouch; excess film is trimmed from the sides, leaving a skirt on the sides and bottom to prevent electrical arcing around the actuator during operation. 3) The flexible joint is made from a flexible hinge layer bonded to a two‐piece stiffening layer with much higher mechanical stiffness. An adhesive layer is applied over the transparency to connect the actuator to the joint. 4) The pouch is bonded to the flexible joint and filled with liquid dielectric using a syringe with an angled needle inserted into the filling port. The filling port is then closed by heat sealing. B) The completed SES joint is characterized by the electrode height (*h*), the pouch width (*w*), and the height of the notched region that is not covered by electrodes (*r*). Joints have a natural resting angle, *θ*_0_.

The fabrication process for SES joints is simple and customizable, which allows them to be tailored for desired properties by modifying films, liquid dielectrics, electrodes, hinges, and stiffening layers as described below. The basic procedure relies on the integration of a variable‐stiffness structure with a high‐performance electrohydraulic component, for which the fabrication procedure is based on techniques introduced by Mitchell et al. for HASEL actuators.^[^ ^46^ ^]^ Details are provided in the Supporting Information, while an overview is provided in **Figure** **2**A.

Step 1: Two dielectric films are heat‐sealed together to form a pouch using a CNC‐controlled heat sealer. A fill port is left open for later filling with liquid dielectric. Step 2: Flexible carbon‐based electrodes are printed on both sides of the film using a screen‐printing method. Excess film is trimmed to reduce constraints on actuation, leaving a skirt to prevent electrical arcing around the actuator during application of high voltage. Step 3: A flexible joint is created by bonding a flexible hinge layer to a two‐piece stiffening layer. This stiffening layer can be flexible or rigid. The hinge stabilizes the joint against lateral loading and provides an elastic restoring force. An adhesive layer (e.g., transfer tape) is applied to the joint for mounting the actuator. Step 4: The empty actuator is adhered to the joint, then filled with liquid dielectric through the fill port. A syringe with a bent needle allows access to the fill port while the actuator is mounted. Finally, the fill port is sealed using a heated soldering iron tip.

For SES joints tested in this work, the default hinge layer was a 75 µm thick transparency with adhesive on one side, for bonding to the stiffening layer. The default stiffening layer was acrylic that was laser cut into shape.

A schematic of a completed joint is shown in Figure 2B. Actuator dimensions are characterized by *h* × *w* × *r*, where *h* is the height of the electrodes in cm, *w* is the width of the pouch (and electrodes) in cm, and *r* is the height of the notched region of the pouch, uncovered by electrodes, in cm. Completed joints have a natural resting angle, *θ*_0_, of 10°–15° (under no load) that results from the deformation caused by mounting the actuators empty then filling with liquid dielectric (Figure 2B).

### 2.2 Quasi‐Static Actuation Performance

#### 2.2.1 High Torque Production

The torque output of SES joints was measured as a function of hinge angle, *θ*. Actuators with various dimensions, *h* × *w* × *r* (Figure 2B), were measured using two types of film: 18 µm thick biaxially oriented polypropylene (BOPP) and 20 µm thick polyester film, brand name L0WS. L0WS was tested as a higher permittivity film (measured *ε*_r_ =  3.15, see Supporting information) that should have better overall performance compared to BOPP (*ε*_r_ =  2.2^[^ ^56^ ^]^). The liquid dielectric used was an ester‐based transformer oil called Envirotemp FR3, with fill amounts for each pouch given in Table S1 of the Supporting Information. A custom setup was used to measure torque (Figure S1, Supporting Information). Details of this setup and the measurement procedure are provided in the Supporting Information. The testing voltage was a modified square wave with amplitude 9 kV (Figure S1E, Supporting Information).

**Figure 3:**
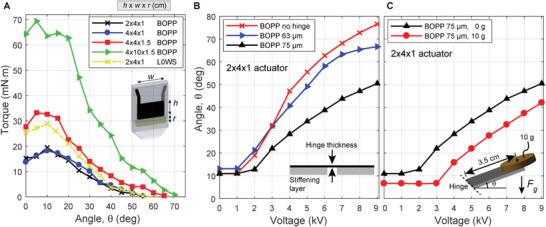
Evaluation of quasi‐static actuation performance in SES joints. A) Torque versus angle curves at 9 kV for SES joints with varying pouch geometry (*h* × *w* × *r*) and film material. For the same voltage, using L0WS increased torque output by ≈ 50% (compared to BOPP). B) Angle versus voltage curves for joints with varying hinge thickness (corresponding to hinge stiffness). Less stiff hinges reached higher angles at a given voltage. C) Angle versus voltage curves for joints made from BOPP films with zero‐ and 10 g external loads (3.5 cm away from the hinge). Hinges were tested horizontally, with the direction of the gravitational force denoted by *F*_g_.

Measured torque versus angle curves are shown in **Figure** **3**A for several actuator geometries using BOPP and L0WS films. The measured torque was relatively constant at low angles (at or below the natural resting angle), and at higher angles, it decreased in a roughly linear trend. Torques up to 70 mN m and free angles up to 70° were measured. Comparing the measured torque–angle curves of the different actuator geometries revealed a linear scaling of the output torque with actuator width, *w*. The generated torque also increased with the height of the notched region of the actuator, *r*, which changes the amount of liquid dielectric and therefore the hydraulic coupling. For the same geometry, using L0WS film resulted in torques ≈50% higher than BOPP at the same voltage, due to L0WS’ higher permittivity, which increases the Maxwell stress in the actuator.^[^ ^57^ ^]^ Changing actuator dimensions from 2 × 4 × 1 to 4 × 4 × 1 cm had no demonstrable effect on torque output, as the actuators were filled with nominally the same fluid volume, so the hydraulic coupling was unaffected.

Specific torque (N m kg^−1^) is an important metric for rotational actuators used in robotic design.^[^ ^58^, ^59^ ^]^ For SES joints, the specific torque is independent of the width of the actuator (i.e., the output torque of an SES joint can readily be scaled up by increasing its width). A maximum specific torque of 21.2 N m kg^−1^ was achieved for the 2 × 4 × 1 L0WS joint, only considering the mass of the actuator (1.36 g), or 2.3 N m kg^−1^ if including mass of the entire joint tested (12.6 g); normalizing to the mass of the actuation component alone is the most accurate representation of the specific torque of SES joints as SES joints can easily leverage the mechanical components inherent to the robotic structure as the stiffening layer for articulation. This direct integration at the joint avoids the need for additional mass and complexity in the system. Specific energy (J kg^−1^)—a closely related metric—was calculated by integrating under the torque versus angle curve for an SES joint. The 2 × 4 × 1 L0WS joint demonstrated the highest specific energy, 10.3 J kg^−1^, again only considering the mass of the actuator.

#### 2.2.2 Continuous and Tunable Angular Output

SES joints have a controllable angular output that is determined by the voltage applied to the electrodes. To characterize angle versus voltage curves, the joints were mounted horizontally on an acrylic stand; a laser displacement sensor was mounted to the stand a defined distance from the joint (Figure S2A,B, Supporting Information) and a transform was applied based on the known geometry of the stand to convert measured distance to joint rotation angle *θ* (Equation (S1), Supporting Information). The joints were tested using the same signal used for testing torque (Figure S1E, Supporting Information).

Figure 3B shows the output hinge angle as a function of voltage for 2 × 4 × 1 cm BOPP actuators with FR3 fluid using various thickness (i.e., stiffness) hinges and no external load. Angle versus voltage curves displayed an activation voltage below which no deformation occurred (as observed previously in Peano‐HASEL actuators^[^ ^57^ ^]^). After deformation began, the output angle increased monotonically with a slower‐than‐linear rise. As seen in the plot, hinge thickness influenced the angle versus voltage curve when no external loads were present, with the maximum angle of thin (i.e., soft) hinges being larger. With no elastic hinge, rotation angles of nearly 80° were observed (the actuator pouch was adhered directly to the acrylic supports without a separate hinge layer). The angle versus voltage curves for hinges of various thickness should converge as loads are increased and the stiffness of the hinge becomes negligible; further, thicker hinges act to increase the lateral stiffness of the joints, prevent buckling under large moments, and provide a stronger elastic restoring force; therefore, a 75 µm hinge was chosen for general use. Figure 3C shows the controllable angular output of SES joints as a function of activation voltage using a 2 × 4 × 1 BOPP actuator with FR3 under both 0 and 10 g external loads. In addition to controllable angular output, SES joints exhibited repeatable actuation for over 2000 cycles with less than a 3% change in angular output between the first 25 and last 25 cycles (Figure S3, Supporting Information). The SES joint demonstrated 2800 cycles until failure, which occurred via dielectric breakdown through the heat seal. This failure was caused by gradual damage from repeated electrical discharges through the air, emanating from the leads of the electrodes. Suppressing these discharges through methods such as encapsulation of the electrodes in a dielectric material would likely improve lifetime substantially.

### 2.3 Modeling Quasi‐Static Response

In this section we derive a 2D model for the quasi‐static response of SES joints in order to provide a basis for informing future designs and geometries.

#### 2.3.1 Parameterization of Actuator Geometry

**Figure 4:**
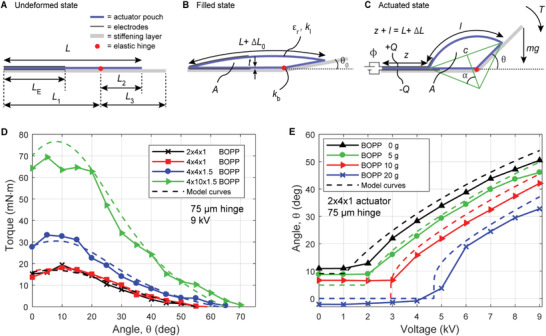
Quasi‐static model of the SES joint. A) Undeformed state: an empty shell (length *L*, width *w* out of the plane of the figure) that is covered on both sides with electrodes (length *L*_E_, width *w* out of the plane of the figure) is bonded to a stiffening layer (assumed to be rigid); a portion (length *L*_1_) attaches to the stationary side, and the remaining portion (length *L*_2_) attaches to the rotating side (length *L*_3_). B) Filled state: when filled with an incompressible liquid dielectric, the cross‐sectional area of the shell (thickness *t*, relative permittivity *ε*_r_) increases to *A* and the hinge (spring constant *k*_b_) rotates by an angle *θ*_0_. Elastic strains in the shell and the joint between shell and stiffening layer (assumed to be rigid) are modeled as an elongation Δ*L*_0_ of the top film of the shell (spring constant *k*_l_). C) When a voltage Φ is applied between the electrodes, they zip together by a length *z* and the hinge rotates to an angle *θ*. The top film elongates by Δ*L*. The unzipped portion of the top film is modeled as a cylinder section of length *l* with central angle 2*α* and chord length *c*. Note that *A* remains constant. We modeled two cases: either a torque *T* or a vertical load *mg* acts on the end of the hinge. D,E) Comparison of model predictions with experimental results.

In this model we distinguish three states (**Figure** **4**A–C). In the undeformed state (Figure 4A), the hinge is flat. It consists of a fixed rigid support (length *L*_1_), and a rotating rigid support (length *L*_3_)—together these make up the stiffening layer for the joint. An empty shell (length *L*, width *w*) is bonded to the flat hinge, so that a portion of length *L*_1_ attaches to the fixed support and the remaining portion ( *L*_2_ =  *L* − *L*_1_) to the rotating support. The shell is covered on both sides with electrodes of length *L*_E_ (= *h* in Figure 2B). In the filled state (Figure 4B), the shell is filled with an incompressible liquid dielectric (volume *V*), which causes the hinge to rotate by an angle *θ*_0_. The cross‐sectional area of the filled shell is *A*  =  *V*/*w*. We treat the top film of the shell as a membrane with negligible bending stiffness, so it takes the shape of a cylinder section.^[^ ^31^ ^]^ To account for the elasticity of the shell and the imperfect connection (subject to delamination) between the shell and the rigid supports we model the top film as extensible (extension Δ*L*_0_, spring constant *k*_l_). The stiffness of the transparency that forms the hinge is modeled as a torsional spring with spring constant *k*_b_. In the zipped state (Figure 4C), a sufficiently large voltage Φ is applied to the electrodes such that they zip together from the edge of the shell over a length *z*, displacing the liquid dielectric and causing the hinge to rotate to an angle *θ*. The elongation of the top film changes to Δ*L* and can be calculated with

(1) $$\Delta L=\frac{c\alpha }{\sin\left(\alpha \right)}+z-L$$

where 2*α* is the central angle of the top film and

(2) $$c=\sqrt{{\left(L_{1}-z+L_{2}\cos\left(\theta \right)\right)}^{2}+L_{2}^{2}\sin{\left(\theta \right)}^{2}\;}$$

is the chord length of the top film. In this state, the cross‐sectional area of the liquid‐filled region of the shell can be calculated with

(3) $$\begin{array}{ccc} A & = & \frac{l^{2}}{8\alpha^{2}}\;\left(2\alpha -\sin\left(2\alpha \right)\right)\\ & & +\;\frac{1}{4}\sqrt{{\left(c^{2}+{\left(L_{1}-z\right)}^{2}+L_{2}^{2}\right)}^{2}-2\left(c^{4}+{\left(L_{1}-z\right)}^{4}+L_{2}^{4}\right)} \end{array}$$

where *l*  =  *L* + Δ*L* − *z* is the length of the cylindrical section of the top film (Figure 4C). Because the liquid dielectric is treated as incompressible, the value of *A* does not change during actuation.

#### 2.3.2 Energy Minimization

To determine the equilibrium position of the electrohydraulic hinge we calculate the extrema of the Helmholtz free energy *F* of the system (the electrohydraulic joint, the voltage source, and an external load). The elastic energy stored in the torsional spring and the shell are (*k*_b_ *θ* ^2^)/2 and (*k*_l_Δ*L* ^2^)/2 respectively. When charges *Q* flow onto the electrodes, the energy of the voltage source reduces by − *Q*Φ. Electrically, the actuator can be treated as a deformable capacitor (capacitance *C*); when charges *Q* flow onto the actuator, the stored electrical energy is *Q* ^2^/2*C*. In this model, we neglect the electric field in the liquid‐filled region of the pouch^[^ ^57^, ^60^ ^]^ and model the zipped region of the electrodes as a parallel plate capacitor with capacitance *C*  =  (*ε*_0_ *ε*_*r*_ *wz*)/2*t*, where *ε*_0_ is the vacuum permittivity, *ε*_r_ is the relative permittivity of the film, *w* is the width of the actuator, *z* is the length of the zipped region of electrodes, and *t* is the thickness of the film that forms the actuator shell (Figure 4). We model two load cases: i) A constant torque *T* is applied to the rotating support. Referenced to the undeformed state, the free energy of the load is *F*_e_  =  *Tθ*. ii) A mass is attached to the end of the rotating support. Referenced to the undeformed state, the potential energy of the load is *F*_e_  =  *mgL*_3_sin (*θ*). The total free energy of the system, thus, becomes

(4) $$\begin{array}{ccc} F\left(Q,z,\alpha ,\theta \right) & = & \frac{1}{2}\;k_{b}\theta^{2}+\frac{1}{2}k_{l}\Delta L{\left(\alpha ,z\right)}^{2}-Q\phi +\frac{1}{2}Q^{2}\frac{2t}{\varepsilon_{0}\varepsilon_{r}wz}\\ & & +\;F_{e}\left(\theta \right) \end{array}$$

We numerically determine the extrema of Equation (4) using the constraint of Equation (3), 0 < *z* < *L_E_* (*z* is limited to be within the electrodes), and *θ* > 0 (contact between the rigid supports prevents bending the hinge to negative angles).

#### 2.3.3 Model Validation

We validated the model for joints made with BOPP film (*t* = 18 µm, *ε*_r_ = 2.2). For pouch width *w* = 4 cm, we estimated *k*_b_ = 4.2 mN m rad^−1^, using material properties of *E ≈* 2 GPa and *ν* ≈ 0.4 for the PET film used in the hinge (Figure S4A,B, Supporting information).^[^ ^61^ ^]^ It is difficult to calculate a value for *k*_l_ so we used it as a fitting factor. Using a value of *k*_l_ = 17.5 N mm^−1^ (determined by visually fitting curves) the calculated torque–angle curves for the BOPP joints agreed very well with the measured torque–angle curves over the entire range of angles (Figure 4D). The model behavior and fit to data is relatively insensitive to small changes in *k*_l_, while model behavior without this fitting factor is unrealistic, predicting asymptotic torque growth at low angles (Figure S5, Supporting Information). For the joint made from 10 cm‐wide BOPP, we estimated *k*_b_ = 9.2 mN m rad^−1^ (see Supporting Information for details) and increased *k*_l_ by a factor of 2.5 to 43.75 N mm^−1^ to account for the larger width. Using this fitting factor, we observed very good agreement between model and experiment for the wider actuator (Figure 4D).

The model explains the shape of the torque–angle curves (load case (i), explained above). When no torque is applied to the joint, the deformation of the joint is only resisted by the bending stiffness of the hinge and the joint achieves the largest angles (if the hinge had no bending stiffness larger angles would be possible (Figure 3B)). With increasing torque, the equilibrium angle decreases until it reaches a maximum at the filling angle *θ*_0_. At *θ*_0_ a snap‐through instability occurs (i.e., the downward portion of the calculated curve for *θ* → 0 represents unstable equilibria). Since the experiment was carried out “angle‐controlled,” the measured torque–angle curve followed the model predictions (in a “torque‐controlled” experiment this region of the torque–angle curve would not be stable). The reason for this snap‐through instability is the elasticity described by *k*_l_.

The model also predicted angle–voltage curves of joints for constant vertical loads very well (load case (ii), Figure 4E). In these calculations, we used the same values for *k*_b_ and *k*_l_ as above and included the torque due to the weight of the rotating support (2.5 g at *L*_3_/2) by adding an equivalent mass of 1.25 g to the external mass, which was located at *L*_3_ from the hinge. The model accurately predicted the presence of an activation voltage, below which no deformation occurred.

### 2.4 Dynamic Actuation Performance

SES joints inherit the fast dynamics of HASEL actuators, which are based on the speed of electrostatics and the local displacement of fluids.^[^ ^62^ ^]^ The nonlinearity of these systems and their tunable response will be consequential for considerations of designs in robotic structures. Therefore, here we explore some of the factors affecting the bandwidth and dynamic characteristics of SES joints.

#### 2.4.1 High Bandwidth Actuation Response

**Figure 5:**
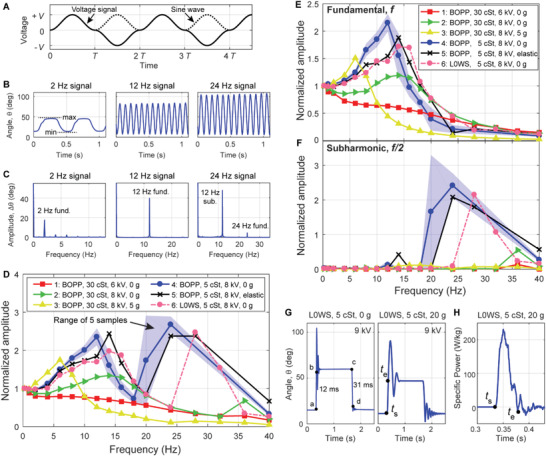
Evaluation of dynamic actuation performance in SES joints. A) The voltage signal used for testing frequency response was a modified sine wave with amplitude *V*. B) Angle versus time responses of a 2 × 4 × 1 BOPP actuator with 5 cSt fluid at 8 kV. Note that the angular response to the 24 Hz signal only had 12 maxima and minima in 1 s. C) The discrete Fourier transforms (DFTs) of the time domain responses in (B) showed a substantial subharmonic resonance when driven by a 24 Hz signal (corresponding to 12 Hz response) due to the nonlinearity of SES joints. D) Frequency response of actuators when analyzed using periodically occurring minimum and maximum values in the time domain (B) to determine amplitude, plotted against the frequency of the driving voltage signal. Various combinations of pouch materials, liquid dielectrics, voltages, and loads were tested. Test 5 added an additional elastic restoring force (Figure S7, Supporting Information). For test 4, the plotted line corresponds to the mean values from five tested samples, while the shaded region is bounded by lowest and highest measured values for those samples. E) Amplitude of the fundamental response (C) plotted against the frequency of the driving voltage signal. F) Amplitude of the subharmonic response (C) plotted against the frequency of the driving voltage signal. G) Response of a 2 × 4 × 1 L0WS joint with 5 cSt fluid to a step voltage at 9 kV, with no load (left) and a 20 g load (right), and H) the corresponding specific power output for a 20 g load.

The bandwidth was tested for SES joints in several different configurations from 0.25 to 40 Hz, using the same experimental setup as angle–voltage measurements (Figure S2, Supporting Information). To identify the effect of material and testing parameters on bandwidth, actuators with dimensions 2 × 4 × 1 cm were fabricated using two types of film (18 µm BOPP and 20 µm L0WS) and liquid dielectrics with two viscosities (FR3 liquid dielectric, *ν* > 30 cSt;^[^ ^63^ ^]^ silicone oil, *ν* = 5 cSt^[^ ^64^ ^]^). One joint was tested with an external load of 5 g (3.5 cm from the hinge) while the remaining joints had no external load. All joints were tested with a modified sine wave (**Figure** **5**A)—one joint was tested at 6 kV, with the remaining tested at 8 kV.

Figure 5B shows the 8 kV angle versus time response of a BOPP joint with 5 cSt fluid for driving signal frequencies of 2, 12, and 24 Hz. From the plots, we see that the joint responds at the same frequency for both the 12 and 24 Hz driving signals. A discrete Fourier transform (DFT) of these responses (Figure 5C) helps explain this unusual behavior—while the joint responds to 2 and 12 Hz signals at the fundamental frequency, the response to the 24 Hz signal occurs primarily at 12 Hz, corresponding to a subharmonic resonance of the system (specifically the second subharmonic, *f*_response_ =  *f*_signal_/2).

The frequency response for several SES joints and test conditions is plotted in Figure 5D, with the *x*‐axis plotting the frequency of the driving signal, and the *y*‐axis plotting the difference between the periodically‐occurring minima and maxima of the angle *θ* (Figure 5B). These amplitudes are normalized to the amplitude of the lowest measured frequency response (0.25 Hz). For all joints using the 5 cSt fluid, a strong resonance peak can be seen between 12 and 14 Hz (corresponding to the fundamental resonance as shown in Figure 5E) with a second larger resonance peak occurring between 24 and 28 Hz (corresponding to a subharmonic resonance as shown in Figure 5F).

To analyze the behavior of the different joints in more detail we took a DFT of the time‐domain response and recorded the amplitude of the fundamental response. Figure 5E plots the amplitude of the response at the fundamental frequency as a function of the frequency of the driving signal (normalized to the response at 0.25 Hz). As shown in Figure 5E, the actuator bandwidth can be controlled by varying voltage, load, liquid dielectric viscosity, and elastic restoring force. Roll‐off frequencies of up to 24 Hz were observed (defined as amplitude = 50% of the 0.25 Hz response amplitude). Several qualitative observations can be made from the data: the BOPP joint with FR3 exhibited flexion angles that decreased monotonically with frequency at 6 kV (test 1), while at 8 kV (test 2) a resonance was present (this behavior is explained in Figure S6, Supporting Information). The resonant behavior was modified by adding an external load of 5 g on the joint (test 3) at the expense of high‐frequency actuation. Using a liquid dielectric with low viscosity (5 cSt silicone oil) led to strong resonance peaks in actuation and better low frequency response, but little change in roll‐off frequency (test 4). Adding a prestrained elastic band (Figure S7, Supporting Information) to the 5 cSt BOPP joint increased the restoring force and improved the roll‐off frequency for the BOPP joint from 19 to 22 Hz (test 5). Finally, using L0WS with low viscosity fluid (test 6) decreased the resonance peak, likely due to the stiffer material, and resulted in a higher roll‐off frequency than the corresponding BOPP joint (test 4). Most tests were performed with one joint; however, test 4 was performed for five identical BOPP, 5 cSt joints to infer data spread for bandwidth tests. The solid line for test 4 in Figure 5D–F represents the mean values from these five samples, while the shaded region represents the range of the data.

Figure 5F plots the second subharmonic amplitudes, normalized to the 0.25 Hz fundamental frequency response. At low frequencies subharmonic response was negligible, but at frequencies above 10 Hz the subharmonic was activated; strong subharmonic response has been observed previously in dielectric elastomer minimum energy structures.^[^ ^65^ ^]^ Figure 5D resembles a superposition of the fundamental (Figure 5E) and second subharmonic (Figure 5F) responses, indicating their relative importance in determining the frequency response of SES joints.

#### 2.4.2 Rapid Actuation Response to a Step Voltage

A fast response to stimuli is important for robotic systems to interact with a dynamic environment. To measure the actuation response of SES joints to a step voltage, we used the experimental setup shown in Figure S2 of the Supporting Information and a square‐wave voltage signal with amplitude 9 kV. Using the material system with the fastest response (L0WS, 5 cSt fluid), the response time was calculated as the time from voltage change—on or off (point a, c)—to the joint reaching 90% of its final resting state (point b, d), Figure 5G (left). The rise times and fall times were 12 and 31 ms, respectively. By comparison, a BOPP joint with FR3 activated at 8 kV demonstrates highly asymmetric response with 28 ms rise time and 298 ms fall time (Movie S5 and Figure S6C, Supporting Information), likely due to the viscosity of the fluid.^[^ ^62^ ^]^

The power output of the L0WS joint was measured using a 20 g load and the same square wave signal at 9 kV. The response was measured from the time of application of voltage (*t*_s_) to equilibrium displacement (*t*_e_), Figure 5G (right). Specific power was calculated using the model in Figure S8 of the Supporting Information by normalizing to the actuator mass, 1.36 g. A maximum specific power of 230 W kg^−1^ was recorded during flexion (Figure 5H; Figure S9, Supporting Information), with an average specific power of 110 W kg^−1^ measured from the initial application of voltage to the equilibrium displacement of the joint—comparable to biological muscle.^[^ ^12^ ^]^ The high specific power allowed for the fabrication of a device capable of jumping 14 times its resting height without the need for power‐amplification mechanisms (Figure 1F).

### 2.5 Power Consumption of an SES Joint versus a Servo Motor

**Figure 6:**
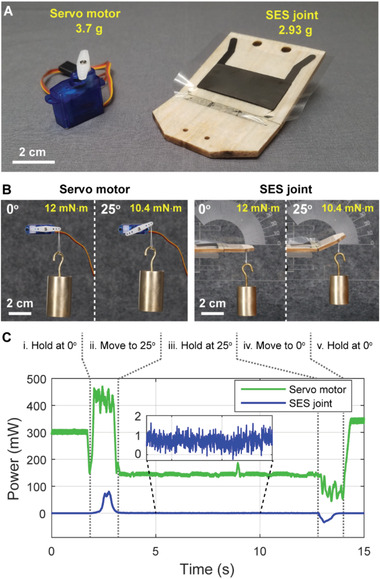
Comparison of power consumption of a servo motor and an SES joint. A) A lightweight SES joint made from balsa wood (2.93 g, peak torque ≈ 30 mN m^−1^ at 9 kV) produced similar maximum torque as a lightweight servo motor (3.7 g without wires, peak torque ≈ 40 mN m at 5 V). B) The weight and lever arm were chosen such that the servo motor and SES joint applied the same torque at all angles. C) Power consumption of both actuators throughout an identical series of motions. The servo motor consumed 140 mW while holding the load at 25° (step iii, (B)), while the SES joint consumed <1 mW.

We compare the power consumption of an SES joint to that of a similarly rated servo motor while undergoing the same sequence of motions. The two actuators used in the comparison were selected for similar weight and peak torque output. An ultralight servo motor weighing 3.7 g (without wires) with peak reported torque around 40 mN m (at 5 V) was used, shown in **Figure** **6**A. We constructed an SES joint using a 2 × 4 × 1 cm actuator with L0WS film and FR3 liquid dielectric attached to a 3 mm balsa wood frame (Figure 6A). Balsa wood was chosen for its high stiffness to weight, leading to a total weight of 2.93 g for the joint. The choice of support material should not directly influence the torque output of the joint, provided it has sufficient stiffness to avoid deflection under applied loads. The 2 × 4 × 1 cm L0WS joint was measured previously to have a peak torque output of around 30 mN m at 9 kV (Figure 3A). Actuators were mounted horizontally to a frame, and a weight was hung from each such that it applied torque of 12 mN m when held at 0° (Figure 6B). The actuators then ran through a preprogrammed cycle: i) hold at 0° for several seconds, ii) move to 25° over 1 s, iii) hold at 25° for 10 s (Figure 6B), iv) move back to 0° over 1 s, and v) hold at 0° for several seconds. To determine power consumption, we monitored voltage and current for the servo motor (experimental details in Figure S10, Supporting Information) and the SES joint (experimental details in Figure S11, Supporting Information).

Power consumption is plotted in Figure 6C. While moving to 25°, the servo motor drew up to 450 mW, while the SES joint drew up to 80 mW. While holding at 25°, the servo motor drew 140 mW continuous power, while the SES joint consumed under 1 mW. The SES joint consumed >80× less power while holding (during step iii) compared to its peak power value while transitioning (in step ii), highlighting that SES joints inherently feature a catch state (consuming negligible power while holding a position). Further, while returning from 25° to 0°, the servo consumed 80 mW, while the power draw of the SES joint was actually “negative”—this energy could be returned to the system through implementation of a charge recovery circuit.^[^ ^66^ ^]^ Since the servo motor was actively holding its position at 0°, it continued to draw power during steps (i) and (v)—mechanical constraints could be implemented to prevent this power consumption in the servo motor while in its “neutral” position of 0°. Additionally, the servo motor could be modified to be non‐backdrivable (creating a low‐energy catch state), but this would increase the weight and complexity of the system and eliminate compliance in the joint.

### 2.6 Robotic Applications: Combining SES Joints for Increased Functionality

**Figure 7:**
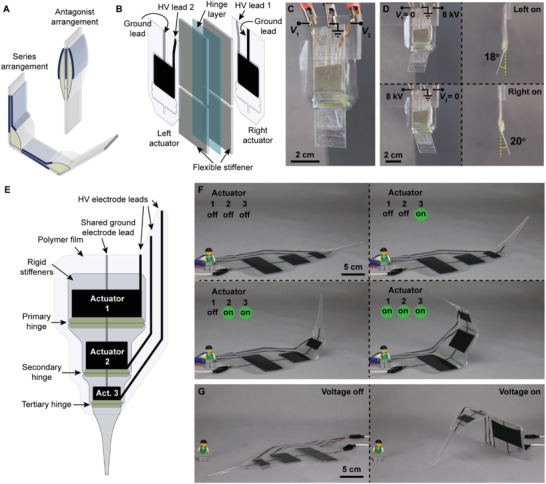
Combining multiple SES joints for different types of motion. A) Combining SES joints into different arrangements enables more diverse actuation: antagonist arrangements allow bidirectional actuation while series arrangements increase angular output. B) A bidirectional SES joint was created by placing one actuator on either side of a bidirectional hinge using transfer tape (not shown). The hinge was made from flexible stiffeners attached to a two‐side adhesive transparency. A gap in the stiffeners allowed for bidirectional actuation in the hinge. C) A bidirectional SES joint made from two 2 × 4 × 1 cm BOPP actuators. Each actuator was independently controlled using *V*_1_ and *V*_2_. D) Activating the left (top) and right (bottom) actuators separately using a voltage of 8 kV resulted in hinge angles of 18° and 20°, respectively. E) By placing several SES joints in series, the overall flexion angle was increased, creating an artificial limb with independently addressable joints. F) Three SES joints activated sequentially with voltages of 8 kV. G) When the actuators were facing downward, the limb could lift itself off the ground.

Many applications of soft robots require systems of independently controlled actuators.^[^ ^67^, ^68^ ^]^ Here, we present two methods of combining SES joints to increase the functionality of these soft robotic devices—antagonist arrangements that allow bidirectional actuation and series arrangements that increase flexion angles (**Figure** **7**A). Details on fabrication of these devices are found in the Supporting Information.

#### 2.6.1 Antagonistic SES Joints for Bidirectional Actuation

To demonstrate the potential for bidirectional actuation, we created a joint made from antagonistic actuator pairs coupled to a bidirectional hinge. Figure 7B shows the basic structure of this joint. Two 2 × 4 × 1 BOPP actuators with FR3 liquid dielectric are placed on either side of a bidirectional hinge made from flexible film stiffeners attached to a two‐side adhesive transparency. A completed bidirectional joint is shown in Figure 7C. The actuators are designed such that they share a common inner electrode, while the outer electrodes can be operated independently. Using a custom H‐bridge circuit, we can selectively activate the left and the right actuators during cycling (Figure S12A–E, Supporting Information). Figure 7D shows two steps of this cycle for powering the left actuator (top) and the right actuator (bottom). This joint was capable of nearly ± 20° of actuation, as well as high‐speed movement (Movie S6, Supporting Information) that resembled the thunniform swimming motion used in the tailfin of many fish species, such as the yellowfin tuna^[^ ^69^ ^]^ and could be readily adapted into a bioinspired robotic prototype.^[^ ^70^ ^]^

#### 2.6.2 Series Arrangement for Multijoint Articulation

Toward the goal of creating maneuverable robots with a large number of independently controlled actuators, we demonstrated an artificial spider limb consisting of three independently controlled SES joints in series. The limb was designed with a tapered structure, Figure 7E, with the largest actuator positioned at the base of the limb to support the additional torque required to lift the subsequent actuators. The actuators shared a ground connection, but had independent high voltage leads that were activated using a three‐channel high voltage power supply.^[^ ^46^ ^]^

Movie S7 (Supporting Information) and Figure 7F show discrete and sequential actuation of each joint with a maximum applied voltage of 8 kV; under no load the limb was able to reach nearly 180° of flexion. Movie S7 of the Supporting Information also shows nonsequential activation of the actuators using both step and ramped voltage signals. Inverting the structure, the artificial spider limb was able to easily lift itself, Figure 7G. Modulating the output voltage using a pressure‐sensitive input device allowed for fast and lifelike actuation, resembling a biological system (Movie S7, Supporting Information).

#### 2.6.3 A Versatile and Strong Gripper

**Figure 8:**
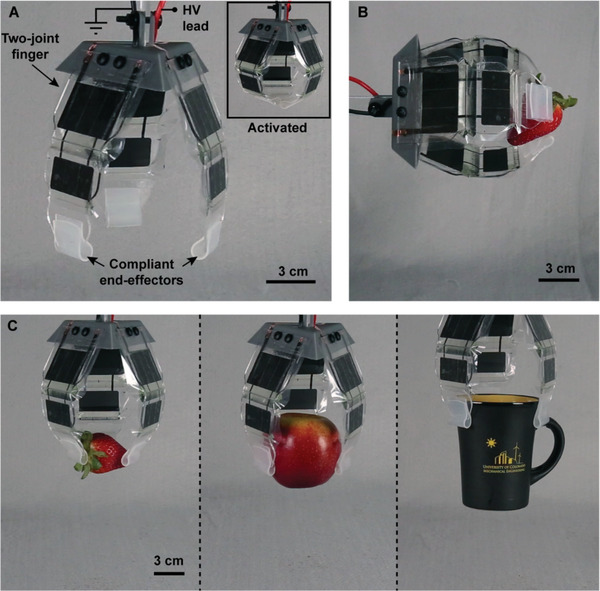
A three‐finger gripper based on series arrangements of SES joints. A) The gripper used three fingers with two joints each. These fingers were actuated simultaneously to perform gripping tasks. Compliant pads coated with a silicone elastomer increased contact area and friction. The gripper closed entirely under application of an 8 kV DC voltage (inset). B) SES joints had sufficient mechanical stability to enable horizontal gripping of lightweight objects such as a strawberry. A voltage of 6 kV was used. C) The compliance of each finger enabled the gripper to grasp a variety of objects without the need for feedback, including a delicate strawberry (18 g), an apple (170 g), and a ceramic mug (270 g). A voltage of 8 kV was used for all three objects.

The combination of both rigid and soft structures allows for effective force transmission through rigid layers but incorporates compliance through soft layers. This combination has previously demonstrated increased performance in pneumatic grippers versus fully soft designs.^[^ ^71^ ^]^ We built a three‐finger gripper to exemplify the benefits of articulated designs compared to previous implementations of grippers using HASEL actuators. This gripper employed three “fingers,” each with two joints, terminating in a compliant end effector (**Figure** **8**A). This deformable end effector increased the contact area when picking up objects and provided a high friction interface compared to the bare acrylic of the SES joints. Two actuators (one at each joint) were used; they were made from L0WS film with FR3 liquid dielectric, with dimensions of 3 × 5 × 1.5 and 2 × 4 × 1 cm (Figure S13, Supporting Information). They were attached to a rigid acrylic stiffening layer. This gripper was able to “pick” a strawberry in the horizontal orientation when activated at 6 kV (Figure 8B; Movie S7, Supporting Information). In the vertical orientation it was able to grasp objects of various sizes and weights without the need for sensory feedback, always using the same 8 kV voltage signal for grasping. Objects included a strawberry (18 g), an apple (170 g), and a ceramic mug (270 g) (Figure 8C; Movie S8, Supporting Information). Previous designs of curling HASELs with continuum deformation^[^ ^46^, ^55^ ^]^ were only able to lift lightweight objects (table tennis ball ≈3 g, chip bag ≈50 g) due to limitations in stability and strength.

## 3 Conclusion

In this paper we introduce SES joints as a new actuating mechanism for use in soft articulated robots. These spider‐inspired electrohydraulic joints demonstrate high specific torque and bandwidth, low power consumption, and soft actuation (e.g., compliance), all in a low profile and lightweight design—qualities that have been identified as critical in the design of legged robots.^[^ ^58^ ^]^

The high torque output demonstrated by SES joints in this paper stems from the use of highly heterogeneous mechanical structures with discrete regions of stiff support coupled to compliant hinges. This approach leverages principles from design and fabrication techniques^[^ ^72^, ^73^, ^74^ ^]^ that have led to numerous successful robots using bioinspiration to achieve high performance at the mesoscale^[^ ^75^, ^76^, ^77^ ^]^ in the past few decades. Such discretization increases stability and allows for efficient force transmission along the structure, which results in high torque output across a large angular excursion compared to previous designs of electrostatic actuators using bending modes of actuation: dielectric elastomer minimum energy structures have only demonstrated torque outputs of 2.3 mN m^[^ ^43^ ^]^ compared to > 70 mN m for SES joints. Further, compared to continuum curling‐type HASELs,^[^ ^46^, ^55^ ^]^ a gripper made from SES joints was able to demonstrate substantially increased stability and grasp strength for more versatile operation.

When considering specific torque (N m kg^−1^)—an important metric for rotational actuators used in robotic design^[^ ^58^, ^59^ ^]^—SES joints are comparable to electromagnetic actuators, which are the gold standard for cm‐scale robotic systems; we measured specific torques up to 21.2 N m kg^−1^ when normalizing to the mass of the actuator alone (1.36 g). The maximum specific torque across a range of servo motor designs is ≈15 N m kg^−1^, independent of servo size, as demonstrated by Dermitzakis et al.^[^ ^59^ ^]^ Servo motors have the advantage of high torque output across a large rotation angle, but methods for improving the torque output of SES joints could be explored. These methods include the identification of high performance, high permittivity materials, as well as scaling principles that increase the specific energy of SES joints actuators, similar to ones identified previously for Peano‐HASEL actuators.^[^ ^57^ ^]^ Along with these efforts, limitations on scaling of actuator size must be investigated in more detail to identify and expand the useful size range for these electrohydraulic joints for adoption toward spider‐scale robots.^[^ ^36^ ^]^ The effects of material stiffness^[^ ^57^ ^]^ and instabilities in electrohydraulic actuation^[^ ^78^ ^]^ must be characterized and mitigated to ensure robust operation across different length scales.

SES joints are fast, with measured roll‐off frequencies of 24 Hz and activation times as low as 12 ms. Compared to thermally driven systems,^[^ ^33^, ^34^ ^]^ which rely on the slow diffusion of heat in and out of a system, the electrostatic mechanism used in this paper is both fast and scalable to large arrays. SES joints also locally displace fluid within the pouch, which avoids the losses and limitations on bandwidth that are introduced by transporting fluids through supply lines and valves.^[^ ^79^ ^]^ The activation and relaxation times of SES joints (12 ms rise, 31 ms fall) are also substantially reduced compared to curling‐type HASEL designs (>80 ms rise, >1 s fall)^[^ ^46^ ^]^ due to the increased elastic restoring force and reduced flow resistance of the shorter, segmented pouches used in SES joints. The rich nonlinearities observed in the dynamics of SES joints present opportunities for further study, such as the effects of viscosity of the liquid dielectric and external load,^[^ ^62^ ^]^ as well as the elastic restoring force of the hinge; all of these factors play a role in the dynamic behavior of SES joints. Modeling efforts, analogous to ones conducted for Peano‐HASELs,^[^ ^62^ ^]^ could inform scaling principles for SES joints to improve dynamic response.

For untethered robots with onboard energy sources (such as batteries or compressed fluid), high‐efficiency actuators enable fast, extended operation. The electrohydraulic mechanism on which SES joints are based has shown efficiencies of 20% in prototypical systems.^[^ ^44^, ^46^ ^]^ Further, SES joints exhibit a catch state, which means that they consume little to no power while holding an actuation state, with 80× reduced power consumption when holding an actuation state versus transitioning between states. SES joints consumed 140× less power while maintaining the same constant torque output when compared to an off‐the‐shelf servo motor of similar weight; while the large power consumption of the servo was likely attributable to inefficient control electronics, the design of servo motors does not inherently allow for low power catch states. The inherent catch state of SES joints will aid in creating efficient, multi‐DOF robotic systems by allowing for minimal power draw from joints that do not actuate continuously (e.g., stabilizing components). Further, electrostatic actuators can increase apparent efficiencies by implementing charge recovery systems, which have been used previously in piezoelectric actuators to recover over 50% of the energy used during actuation.^[^ ^66^ ^]^

SES joints present several additional characteristics that encourage their use in robotic systems. Compared to traditional fluidic actuation, SES joints eliminate the need for fluid lines and control valves, instead replacing them with in‐plane electrical connections that can be easily patterned. The simplicity and in‐plane design supports the creation of more complex structures by allowing direct integration of actuation at the joint, with minimal peripheral components. SES joints could be applied to a variety of articulating robotic designs, leveraging the mechanical components inherent to the robotic structure to provide the necessary support layer for articulation. Further, their electrical operation is silent and allows for direct electrical‐to‐mechanical control of the actuator output. Control of electrohydraulic systems can be augmented through closed‐loop control methods using external sensors^[^ ^80^ ^]^ or even by exploiting their inherent ability to self‐sense deformation states using capacitance measurements.^[^ ^44^, ^81^ ^]^ Finally, the versatile and facile fabrication process presented here enables the creation of SES joints using numerous materials and geometries for tailored performance. Despite their simplicity, our current process for fabrication experienced issues with delamination of the hinge from the stiffening layer during high torque output at low angles, and after continuous high frequency operation. New methods for integrating these layers, including strategies for monolithic fabrication^[^ ^82^ ^]^ could be explored to increase the reliability of joints. Additionally, designs using functionally graded stiffness could enable more flexible and robust robotic structures.^[^ ^2^, ^83^ ^]^

The family of SES joints introduced here represents a design strategy that closely integrates soft actuation with the mechanical structure of articulated robotic systems. The result is an electrically controlled joint with well‐rounded performance, capable of high forces, high speed operation, with low power consumption in a low‐profile design. With continued research, we hope that SES joints will be versatile building blocks for highly capable spider‐inspired robotic systems that feature novel locomotion and manipulation capabilities.

## 4 Experimental Section

### Fabrication of SES Joints

The fabrication method for the electrohydraulic components used in this work was modified from a rapid prototyping method developed by Mitchell et al.^[^ ^46^ ^]^ Actuators were made from two dielectric polymer films: 1) 18 µm thick BOPP film (5020 film, 70 Ga, Multiplastics Inc.) or 2) 20 µm thick polyester film (L0WS, 80 Ga., Multiplastics Inc.). Electrodes (CI‐2051, Engineered Materials Systems, Inc.) were deposited on the films using a screen‐printing method. Stiffening layers were made from laser‐processed acrylic (Trotec Speedy 360 Flexx, 75W CO_2_). The acrylic was 3 mm thick for torque versus angle tests for rigidity, and 1.5 mm thick for angle versus voltage tests and all dynamic testing. The hinges used in joint characterization were made from adhesive transparency, either 75 µm thick or 63 µm thick (Grafix Light Weight Laminating Film). Adhesive transfer tape (3M, 924) was applied to the stiffening layer to attach the actuator. The use of separate transfer tape allowed precise control over where the actuator was bonded to the joint to prevent overconstraining the system. Liquid dielectric with viscosities of ≈30 cSt (Envirotemp FR3, Cargill) or 5 cSt (silicone oil, 317667, Sigma‐Aldrich) was used. The final fill amount was determined by weight according to Table S1 of the Supporting Information, and measured with a precision balance (Ohaus Adventurer, S05015). Electrical connections to the actuator were made with copper tape (1/8”, Oubaka) with the connection reinforced with conductive carbon glue (Conductive Carbon Glue 16050, PELCO).

### Testing Methods

An NI DAQ (Model USB‐6212 BNC) took voltage signals generated by custom Labview VIs (version 15.0.1f2) or Matlab scripts (2019b) and fed them into a Trek 50/12 high voltage amplifier. Torque versus angle measurements were made using a custom test setup with actuators oriented vertically (Figure S1A, Supporting Information). Torque was measured using a load cell (Robotshop RB‐SEE‐198) via a measurement interface (Figure S1B,C, Supporting Information). A Wheatstone bridge (Phidgets PhidgetBridge, 1046_0B) measured the signal from the load cell using a custom python script (Python 3.7). For angle versus voltage, bandwidth, impulse, and power measurements, angle was measured indirectly using a laser displacement sensor (Keyence, LK‐H057) mounted to a test stand with known geometry to allow transformation of distance to angle (Figure S2, Supporting Information). Laser displacement data for frequency tests was recorded at a rate > 50× actuation frequency, while impulse testing used a fixed 5 kHz sampling rate. Power consumption tests used a lightweight servo motor (Pololu #1053, Sub‐Micro Servo 3.7 g); servo motor power consumption was measured using the circuit in Figure S10 of the Supporting Information. The motor was powered using a 5 V benchtop DC power supply (Keysight U8002A) and controlled using the PWM signal from a microcontroller (Elegoo Mega 2560) and the “Servo” control library in Arduino (version 1.8.1). An NI DAQ (Model USB‐6212 BNC) sampling at 200 kHz monitored the voltage across both the motor and a 206 mΩ measuring resistor that was used to determine current. Power consumption of the SES joint was measured using the circuit in Figure S11 of the Supporting Information. A current meter (uCurrent Gold rev. 2) measured the current provided by the power supply while the voltage output to the SES joint was measured using the voltage monitor from the Trek 50/12. Both signals were recorded by an NI DAQ (USB‐6212 BNC) at 10 kHz.

### Bidirectional SES Joint

Pouches were 2 × 4 × 1 cm using BOPP film. To make the support structure, a two‐side adhesive film (Grafix Double Tack Mounting Film) was used as the elastic hinge material. Flexible stiffening layers were transparency film with thickness 125 µm (Grafix Heavy Weight Laminating Film). Actuators used conductive mesh tape for the electrodes attached to the joint (*XYZ*‐axis, 9719, 3M), and an electrically conductive hydrogel^[^ ^84^ ^]^ swelled with LiCl aqueous solution^[^ ^85^ ^]^ for the outer electrodes, prepared according to Kellaris et al.^[^ ^57^ ^]^ Actuators were mounted using adhesive transfer tape (3M, 924). Since the actuators are not stretchable, slack was built into the system by biasing the hinge away from the actuator being mounted by 45° before attaching the actuator to the lower portion of the support. Bidirectional SES joints were controlled using the circuit shown in Figure S12 of the Supporting Information. Optocouplers (OC100HG, Voltage Multipliers Inc.) were used to switch HV connections. The LEDs of each optocoupler were operated using the circuit presented by PetaPicoVoltron.^[^ ^86^ ^]^ This driving scheme was designed to reverse the polarity of the voltage applied to each actuator during subsequent actuation cycles.

### Artificial Spider Limb

The high‐voltage electrodes and leads of the actuators were coated with a 200 µm thick layer of silicone elastomer (Ecoflex 00–30, Smooth‐On) to prevent electrical arcing during independent operation. The stiffening layer was 1.5 mm acrylic and hinges were the 75 µm transparency. The joints were driven independently using the three‐channel power supply created previously by Mitchell et al.^[^ ^46^ ^]^ When operating the actuators simultaneously, a pressure‐sensitive resistor (SEN‐09375, SparkFun) was used to modulate voltage output proportional to force.

### Three‐Finger Gripper

Each finger was comprised of two SES joints made from 20 µm L0WS film with pouch dimensions 3 × 5 × 1.5 cm and 2 × 4 × 1 cm (Figure S13A, Supporting Information). The stiffening layer was 1.5 mm acrylic (Figure S13C, Supporting Information). A compliant end effector was added to the end of each finger and was made from a simple strip of transparency film, looped over the end of the acrylic and adhered at the ends using the adhesive transfer tape. This was covered by a 0.5 mm thick strip of elastomer (Ecoflexx 00–30) to provide a high‐friction interface. The gripper base was tapered by 15° to bias the fingers outward for larger grasps.

### Statistical Analysis

Experimental results presented in this work used *n* = 1 (a single SES joint) unless otherwise indicated. For measuring torque versus angular output, voltage was applied to the SES joint for several cycles—the peak torque output was measured for each cycle and averaged across four cycles to produce the reported torque value at each angle. No filtering was performed in the data. Angle versus voltage tests used single SES joints, with the voltage applied for several cycles. The maximum angular output was measured for each cycle and averaged over several cycles. For the measured permittivity of the L0WS film, the capacitance of nine samples with known geometry was measured to calculate permittivity. The average relative permittivity was reported. For frequency response tests, discrete frequencies were tested and the angular output at each frequency was reported. Laser displacement data were recorded at a rate > 50× the applied actuation signal to determine angular output. Data were recorded for ≈10 s to allow for transient actuation effects to die off. Two methods of analysis were used for the data: first, a Fourier transform was performed for the data range that did not exhibit transient effects with the fundamental and second subharmonic amplitudes recorded and plotted. These were normalized to the amplitude measured for the lowest tested frequency for each SES joint (0.25 Hz). Second, the amplitude of the angular output of each SES joint was measured in the time domain and averaged across the data range and reported for each frequency tested. All tests used *n* = 1 with the exception of test 4, which used *n* = 5. For test 4, the reported angular amplitude for each frequency used the mean values across the five samples tested. The shaded area represents the range of the data for the five samples. For power output tests, displacement data for the SES joint was recorded at 5 kHz, then smoothed using a Savitzky–Golay filter with a third order polynomial and a window of 201 points. Velocity and acceleration data were calculated from the filtered displacement data. Power consumption tests for SES joints filtered current data using a Savitzky–Golay filter using a third‐order polynomial fit with a window of 101 points, followed a moving average with a window of 100 points. Power consumption for the servo motor recorded current and voltage data at 200 kHz. The control electronics for the servo motor caused current draw for the motor to vary at high frequencies. To smooth these data to calculate average power, the current data were filtered using a Savitzky–Golay filter with a third‐order polynomial and a window of 5001 points, followed by a moving average with a window of 20k points.

## Conflict of Interest

The authors declare no conflict of interest.

## Author Contributions

C.K. conceived and supervised the research. C.K. and K.J. shaped the vision of the study and K.J. provided biological context. N.K. conceived the design of SES joints and N.K., Y.Z., and G.M.S. fabricated devices. N.K., Y.Z., and G.M.S. designed experimental setups, collected, and analyzed data. P.R. developed the quasi‐static model of SES joints and guided analysis and interpretation of dynamic joint characteristics. S.K.M. developed and characterized the artificial limb, created the control electronics for the bidirectional joint, and provided measurements of the permittivity of L0WS film. N.K., P.R., and S.K.M. drafted and revised the manuscript and figures, with guidance from C.K. and K.J.

## Competing Interests

N.K., S.K.M., and C.K. are listed as inventors on patent applications PCT/US18/023797 and PCT/US19/020568 that cover fundamentals and basic designs of HASEL actuators. N.K., S.K.M., and C.K. are cofounders of Artimus Robotics, a start‐up company commercializing electrohydraulic HASEL actuators.

## Supporting information

Supporting Information

Supplemental Movie 1

Supplemental Movie 2

Supplemental Movie 3

Supplemental Movie 4

Supplemental Movie 5

Supplemental Movie 6

Supplemental Movie 7

Supplemental Movie 8

## Data Availability

All data necessary to evaluate the conclusions of this work are available in the paper or the Supporting Information.

## References

[1] H. Yuk , S. Lin , C. Ma , M. Takaffoli , N. X. Fang , X. Zhao , Nat. Commun. 2017, 8, 14230. 28145412 10.1038/ncomms14230 PMC5296644

[2] K. Kumar , J. Liu , C. Christianson , M. Ali , M. T. Tolley , J. Aizenberg , D. E. Ingber , J. C. Weaver , K. Bertoldi , Soft Rob. 2017, 4, 317. 10.1089/soro.2017.0002 29251563

[3] M. T. Tolley , R. F. Shepherd , B. Mosadegh , K. C. Galloway , M. Wehner , M. Karpelson , R. J. Wood , G. M. Whitesides , Soft Rob. 2014, 1, 213.

[4] K. Suzumori , S. Iikura , H. Tanaka , 1991 IEEE Int. Conf. on Robotics and Automation Proc., IEEE, Piscataway, NJ 1991, Vol. 2, pp. 1622–1627.

[5] P. Polygerinos , Z. Wang , K. C. Galloway , R. J. Wood , C. J. Walsh , Rob. Auton. Syst. 2015, 73, 135.

[6] Z. Zhakypov , K. Mori , K. Hosoda , J. Paik , Nature 2019, 571, 381. 31292552 10.1038/s41586-019-1388-8

[7] R. Baumgartner , A. Kogler , J. M. Stadlbauer , C. C. Foo , R. Kaltseis , M. Baumgartner , G. Mao , C. Keplinger , S. J. A. Koh , N. Arnold , Z. Suo , M. Kaltenbrunner , S. Bauer , Adv. Sci. 2020, 7, 1903391. 10.1002/advs.201903391 PMC7055565 32154089

[8] D. Trivedi , C. D. Rahn , W. M. Kier , I. D. Walker , Appl. Bionics Biomech. 2008, 5, 99.

[9] S. Kim , C. Laschi , B. Trimmer , Trends Biotechnol. 2013, 31, 287. 23582470 10.1016/j.tibtech.2013.03.002

[10] H. Lipson , Soft Rob. 2014, 1, 21.

[11] D. Rus , M. T. Tolley , Nature 2015, 521, 467. 26017446 10.1038/nature14543

[12] S. M. Mirvakili , I. W. Hunter , Adv. Mater. 2018, 30, 1704407. 10.1002/adma.201704407 29250838

[13] E. R. Trueman , The Locomotion of Soft‐Bodied Animals, Edward Arnold, London 1975.

[14] M. E. G. Evans , J. Zool. 1977, 181, 189.

[15] B. A. Trimmer , H.‐T. Lin , Integr. Comp. Biol. 2014, 54, 1122. 24944114 10.1093/icb/icu076

[16] A. G. Winter , V. R. L. H. Deits , D. S. Dorsch , A. H. Slocum , A. E. Hosoi , Bioinspir. Biomim. 2014, 9, 036009. 24713848 10.1088/1748-3182/9/3/036009

[17] M. Wehner , R. L. Truby , D. J. Fitzgerald , B. Mosadegh , G. M. Whitesides , J. A. Lewis , R. J. Wood , Nature 2016, 536, 451. 27558065 10.1038/nature19100

[18] C. Laschi , M. Cianchetti , B. Mazzolai , L. Margheri , M. Follador , P. Dario , Adv. Rob. 2012, 26, 709. 10.1088/1748-3182/7/2/025005 22617166

[19] R. F. Shepherd , F. Ilievski , W. Choi , S. A. Morin , A. A. Stokes , A. D. Mazzeo , X. Chen , M. Wang , G. M. Whitesides , Proc. Natl. Acad. Sci. USA 2011, 108, 20400. 22123978 10.1073/pnas.1116564108 PMC3251082

[20] R. E. Snodgrass , Principles of Insect Morphology, Cornell University Press, Ithaca, NY 2018.

[21] R. J. Wootton , J. Exp. Biol. 1999, 202, 3333. 10562516 10.1242/jeb.202.23.3333

[22] R. J. Full , M. S. Tu , J. Exp. Biol. 1991, 156, 215. 2051129 10.1242/jeb.156.1.215

[23] K. Jayaram , R. J. Full , Proc. Natl. Acad. Sci. USA 2016, 113, E950. 26858443 10.1073/pnas.1514591113 PMC4776529

[24] Spider Behaviour: Flexibility and Versatility (Ed: M. E. Herberstein ), Cambridge University Press, Cambridge 2011.

[25] J. W. Shultz , Zool. J. Linn. Soc. London 1989, 97, 1

[26] W. G. Eberhard , Proc. R. Soc. B 2007, 274, 2203. 10.1098/rspb.2007.0675 PMC2706203 17609181

[27] B. Eggs , J. O. Wolff , L. Kuhn‐Nentwig , S. N. Gorb , W. Nentwig , Ethology 2015, 121, 1166.

[28] M. R. A. Nabawy , G. Sivalingam , R. J. Garwood , W. J. Crowther , W. I. Sellers , Sci. Rep. 2018, 8, 7142. 29739977 10.1038/s41598-018-25227-9 PMC5940701

[29] S. Landkammer , F. Winter , D. Schneider , R. Hornfeck , Robotics 2016, 5, 15.

[30] A. Nemiroski , Y. Y. Shevchenko , A. A. Stokes , B. Unal , A. Ainla , S. Albert , G. Compton , E. MacDonald , Y. Schwab , C. Zellhofer , G. M. Whitesides , Soft Rob. 2017, 4, 183. 10.1089/soro.2016.0043 29182080

[31] R. Niiyama , X. Sun , C. Sung , B. An , D. Rus , S. Kim , Soft Rob. 2015, 2, 59.

[32] A. Sprowitz , C. Gottler , A. Sinha , C. Caer , M. U. Ooztekin , K. Petersen , M. Sitti , 2017 IEEE Int. Conf. on Robotics and Automation (ICRA), IEEE, Singapore 2017, pp. 64–70.

[33] A. Miriyev , K. Stack , H. Lipson , Nat. Commun. 2017, 8, 596. 28928384 10.1038/s41467-017-00685-3 PMC5605691

[34] X. Huang , M. Ford , Z. J. Patterson , M. Zarepoor , C. Pan , C. Majidi , J. Mater. Chem. B 2020, 8, 4539. 32373836 10.1039/d0tb00392a

[35] N. T. Jafferis , E. F. Helbling , M. Karpelson , R. J. Wood , Nature 2019, 570, 491. 31243384 10.1038/s41586-019-1322-0

[36] K. Jayaram , J. Shum , S. Castellanos , E. F. Helbling , R. J. Wood , *2020 IEEE Int. Conf. on Robotics and Automation Proc*., IEEE, Piscataway, NJ 2020, pp. 10305–10311.

[37] R. Pelrine , R. Kornbluh , Q. Pei , J. Joseph , Science 2000, 287, 836. 10657293 10.1126/science.287.5454.836

[38] I. A. Anderson , T. A. Gisby , T. G. McKay , B. M. O'Brien , E. P. Calius , J. Appl. Phys. 2012, 112, 041101.

[39] Y. Chen , H. Zhao , J. Mao , P. Chirarattananon , E. F. Helbling , N. P. Hyun , D. R. Clarke , R. J. Wood , Nature 2019, 575, 324. 31686057 10.1038/s41586-019-1737-7

[40] X. Ji , X. Liu , V. Cacucciolo , M. Imboden , Y. Civet , A. E. Haitami , S. Cantin , Y. Perriard , H. Shea , Sci. Rob. 2019, 4, 6451. 10.1126/scirobotics.aaz6451 33137720

[41] Y. Gao , X. Fang , D. Tran , K. Ju , B. Qian , J. Li , R. Soc. Open Sci. 2019, 6, 182145. 31598224 10.1098/rsos.182145 PMC6731732

[42] G. Kofod , W. Wirges , M. Paajanen , S. Bauer , Appl. Phys. Lett. 2007, 90, 081916.

[43] J. Zhao , J. Niu , D. McCoul , Y. Ge , Q. Pei , L. Liu , J. Leng , Appl. Phys. Lett. 2015, 107, 063505.

[44] E. Acome , S. K. Mitchell , T. G. Morrissey , M. B. Emmett , C. Benjamin , M. King , M. Radakovitz , C. Keplinger , Science 2018, 359, 61. 29302008 10.1126/science.aao6139

[45] N. Kellaris , V. Gopaluni Venkata , G. M. Smith , S. K. Mitchell , C. Keplinger , Sci. Rob. 2018, 3, 3276. 10.1126/scirobotics.aar3276 33141696

[46] S. K. Mitchell , X. Wang , E. Acome , T. Martin , K. Ly , N. Kellaris , V. G. Venkata , C. Keplinger , Adv. Sci. 2019, 6, 1900178. 10.1002/advs.201900178 PMC6662077 31380206

[47] X. Wang , S. K. Mitchell , E. H. Rumley , P. Rothemund , C. Keplinger , Adv. Funct. Mater. 2020, 30, 1908821.

[48] J.‐P. Hubschman , J.‐L. Bourges , W. Choi , A. Mozayan , A. Tsirbas , C.‐J. Kim , S.‐D. Schwartz , Eye 2010, 24, 364. 19300461 10.1038/eye.2009.47

[49] L. Belding , B. Baytekin , H. T. Baytekin , P. Rothemund , M. S. Verma , A. Nemiroski , D. Sameoto , B. A. Grzybowski , G. M. Whitesides , Adv. Mater. 2018, 30, 1704446. 10.1002/adma.201704446 29334140

[50] C. Göttler , K. Elflein , R. Siegwart , M. Sitti , Adv. Sci. 2021, 8, 2003890. 10.1002/advs.202003890 PMC7927609 33717859

[51] R. Niiyama , D. Rus , S. Kim , 2014 IEEE Int. Conf. on Robotics and Automation (ICRA), IEEE, Piscataway, NJ 2014, pp. 6332–6337.

[52] R. Blickhan , F. G. Barth , J. Comp. Physiol., A 1985, 157, 115.

[53] R. F. Foelix , Biology of Spiders, Oxford University Press, Oxford 2011.

[54] A. T. Sensenig , J. Exp. Biol. 2003, 206, 771. 12517993 10.1242/jeb.00182

[55] T. Park , K. Kim , S.‐R. Oh , Y. Cha , Soft Rob. 2020, 7, 68. 10.1089/soro.2019.0009 31549923

[56] “Comparative data for plastic films | Mitsubishi Polyester Film GmbH,” can be found under https://www.m‐petfilm.de/en/service/comparative‐data‐for‐plastic‐films/ (accessed: June 2020).

[57] N. Kellaris , V. G. Venkata , P. Rothemund , C. Keplinger , Extreme Mech. Lett. 2019, 29, 100449.

[58] P. M. Wensing , A. Wang , S. Seok , D. Otten , J. Lang , S. Kim , IEEE Trans. Rob. 2017, 33, 509.

[59] K. Dermitzakis , J. P. Carbajal , J. H. Marden , Proc. Comput. Sci. 2011, 7, 250.

[60] G. Moretti , M. Duranti , M. Righi , R. Vertechy , M. Fontana , Proc. SPIE 2018, 10594, 105940W.

[61] Springer Handbook of Condensed Matter and Materials Data (Eds: W. Martienssen , H. Warlimont ), Springer, Heidelberg 2005.

[62] P. Rothemund , S. Kirkman , C. Keplinger , Proc. Natl. Acad. Sci. USA 2020, 117, 16207. 32601189 10.1073/pnas.2006596117 PMC7368252

[63] “FR3 Natural Ester Fluid Technical Details | Cargill,” can be found under https://www.cargill.com/bioindustrial/fr3‐fluid/fr3‐fluid‐technical‐details (accessed: June 2020).

[64] “Silicone oil 317667,” can be found under https://www.sigmaaldrich.com/Graphics/COfAInfo/SigmaSAPQM/SPEC/31/317667/317667‐BULK_______ALDRICH__.pdf (accessed: June 2020).

[65] H. Vatanjou , Y. Hojjat , M. Karafi , Appl. Phys. A 2019, 125, 583.

[66] D. Campolo , M. Sitti , R. S. Fearing , IEEE Trans. Ultrason. Ferroelectr. Freq. Control 2003, 50, 237. 12699157 10.1109/tuffc.2003.1193617

[67] S. I. Rich , R. J. Wood , C. Majidi , Nat. Electron. 2018, 1, 102.

[68] J. M. McCracken , B. R. Donovan , T. J. White , Adv. Mater. 2020, 32, 1906564. 10.1002/adma.201906564 32133704

[69] R. E. Shadwick , D. A. Syme , J. Exp. Biol. 2008, 211, 1603. 18456888 10.1242/jeb.013250

[70] Z. Wolf , A. Jusufi , D. M. Vogt , G. V. Lauder , Bioinspir. Biomim. 2020, 15, 046008. 32330908 10.1088/1748-3190/ab8d0f

[71] Z. Wu , X. Li , Z. Guo , Chin. J. Mech. Eng. 2019, 32, 78.

[72] R. Merz , F. B. Prinz , K. Ramaswami , M. Terk , L. E. Weiss , 1994 Int. Solid Freeform Fabrication Symp., University of Texas at Austin, Austin, TX 1994.

[73] R. J. Wood , S. Avadhanula , R. Sahai , E. Steltz , R. S. Fearing , J. Mech. Des. 2008, 130, 052304.

[74] J. P. Whitney , P. S. Sreetharan , K. Y. Ma , R. J. Wood , J. Micromech. Microeng. 2011, 21, 115021.

[75] J. G. Cham , S. A. Bailey , J. E. Clark , R. J. Full , M. R. Cutkosky , Int. J. Rob. Res. 2002, 21, 869.

[76] S. Kim , J. E. Clark , M. R. Cutkosky , Int. J. Rob. Res. 2006, 25, 903.

[77] K. Y. Ma , P. Chirarattananon , S. B. Fuller , R. J. Wood , Science 2013, 340, 603. 23641114 10.1126/science.1231806

[78] P. Rothemund , N. Kellaris , C. Keplinger , Extreme Mech. Lett. 2019, 31, 100542.

[79] S. Davis , J. Canderle , P. Artrit , N. Tsagarakis , D. G. Caldwell , in Proc. 2002 IEEE Int. Conf. on Robotics and Automation, Cat. No. 02CH37292, Vol. 3, IEEE, Piscataway, NJ 2002, pp. 2836–2841.

[80] B. K. Johnson , V. Sundaram , M. Naris , E. Acome , K. Ly , N. Correll , C. Keplinger , J. S. Humbert , M. E. Rentschler , IEEE Rob. Autom. Lett. 2020, 5, 3783.

[81] K. Ly , N. Kellaris , D. McMorris , B. K. Johnson , E. Acome , V. Sundaram , M. Naris , J. S. Humbert , M. E. Rentschler , C. Keplinger , N. Correll , Soft Rob. 2020. 10.1089/soro.2020.0048 33001742

[82] T. Ranzani , S. Russo , N. W. Bartlett , M. Wehner , R. J. Wood , Adv. Mater. 2018, 30, 1802739. 10.1002/adma.201802739 30079470

[83] N. W. Bartlett , M. T. Tolley , J. T. B. Overvelde , J. C. Weaver , B. Mosadegh , K. Bertoldi , G. M. Whitesides , R. J. Wood , Science 2015, 349, 161. 26160940 10.1126/science.aab0129

[84] C. Keplinger , J.‐Y. Sun , C. C. Foo , P. Rothemund , G. M. Whitesides , Z. Suo , Science 2013, 341, 984. 23990555 10.1126/science.1240228

[85] Y. Bai , B. Chen , F. Xiang , J. Zhou , H. Wang , Z. Suo , Appl. Phys. Lett. 2014, 105, 151903.

[86] S. Schlatter , P. Illenberger , S. Rosset , HardwareX 2018, 4, 00039.

