# Supplementary material for: Spider‐Inspired Electrohydraulic Actuators for Fast, Soft‐Actuated Joints
Source: Adv Sci (Weinh). 2021 May 29;8(14):2100916. doi: 10.1002/advs.202100916 (PMC8292915; doi:10.1002/advs.202100916)
Supplement: Supplementary file 1 — Supporting Information [file ADVS-8-2100916-s008.pdf]

© 2021 Wiley-VCH GmbH

ADVANCED SCIENCE
Open Access

Supporting Information

for *Adv. Sci.,* DOI: 10.1002/advs.202100916

Spider-inspired electrohydraulic actuators for fast, soft-actuated joints

Nicholas Kellaris, Philipp Rothemund, Yi Zeng, Shane K. Mitchell, Garrett M. Smith, Kaushik Jayaram, and *Christoph Keplinger**

Supporting Information

Title: Spider-inspired electrohydraulic actuators for fast, soft-actuated joints

*Nicholas Kellaris, Philipp Rothemund, Yi Zeng, Shane K. Mitchell, Garrett M. Smith, Kaushik Jayaram, Christoph Keplinger**

Materials and Methods

Fabrication of SES joints

Sealing films into pouch: Dielectric polymer films were made from either 18-µm-thick biaxially-oriented polypropylene (BOPP) film from Multiplastics Inc. (5020 film, 70 Ga.) or 20-µm-thick polyester film (L0WS, 80 Ga., Multiplastics Inc.). These films had a heat sealable layer on one side and a hydrophilic layer on the other side to promote ink adhesion. The films were sealed together using a CNC machine (Carbide 3D Shapeoko XL) with a heated 3D printing head and a custom brass tip. A 1/16"-thick neoprene rubber sheet was placed over the CNC bed to distribute pressure from the brass tip. A 1-mil (0.001") layer of Kapton film (DuPont) was placed over the neoprene to prevent film layers from sticking to the neoprene during sealing. The two layers of film were placed on the Kapton with heat sealing layers inward and covered with 1-mil Kapton film (DuPont) to protect them from abrasion and overheating while sealing. A tip temperature of 195 ^{o}C was used, along with a sealing speed of 500 mm/min for BOPP and 400 mm/min for L0WS. The pouch shapes were designed using CAD software (Solidworks 2019), exported as a .dxf file, and converted to g-code format using open-source software (dxf2gcode). The pouch shapes were designed to include a filling port to allow them to be filled with liquid (**Figure 2A**).

Geometry of pouches: Pouch geometries are referred to by their dimensions *h* x *w* x *r*, where *h* is the height of the area to be covered in electrodes in cm, *w* is the width of the pouch in cm, and *r* is the height of the notched region of the pouch that is uncovered by electrodes, in cm. The notched shape in the actuators was introduced by Kellaris et al.[57] and is designed to facilitate buckling at the pouch sides to enable the pouch to form a cylindrical section with minimal side constraints.

Fabrication of the electrodes: Electrodes were deposited on the films using a screen-printing method. A polyester screen with a mesh of 200 threads/cm (Gold-Up USA) was used. A UV-curable

emulsion (Proclaim Dual Cure, Ulano) was deposited on both sides of the screen and allowed to dry for > 4 hours in a dark room. Electrode patterns were created in CAD software (Solidworks 2019) and printed on a transparency to create a positive of the electrode pattern. These transparencies were placed onto the screen and exposed to 365 nm UV light (Analytik Jena, XX-40) for 10 minutes to cure the emulsion exposed to light. The region covered by the transparency pattern was rinsed off with water, creating a negative of the electrode pattern.

To print electrodes onto the pouches, the screen was mounted to hinges to fix its alignment over a glass plate. The electrode pattern was printed onto the glass plate as an alignment guide when printing on pouches. To print on pouches, the sealed films were placed on the glass plate and the screen was rotated off the surface and flooded with conductive carbon ink (CI-2051, Engineered Materials Systems, Inc.). The screen was lowered over the surface of the films and an 85A durometer squeegee (Gold-Up USA) was dragged across the screen with constant pressure to transfer the ink to the films. The ink was allowed to fully dry in ambient conditions (~ 8 hours) before electrodes were deposited on the reverse side of the films. After printing, the films were trimmed to remove excess material while leaving a 1-cm skirt around the pouch to prevent electrical arcing during operation.

Electrodes for all actuators were dimensioned to be slightly smaller than the interior dimensions of the pouches (e.g. 4 cm inner pouch width used 3.9 cm electrode width) to provide tolerance for misalignment in screenprinting.

Fabrication of the joint support structure: To create the joint support structure, first a stiffening layer was chosen. Most actuators used an acrylic stiffening layer as acrylic is available in a variety of thicknesses and can be easily processed. Geometries of the stiffening layers were designed in a CAD program (Solidworks 2019), exported to a .dxf file, and then processed with a lasercutter (Trotec Speedy 360 Flexx, 75W CO_2). The top edge of one half of the stiffening layer was joined to the bottom edge of the other half of the stiffening layer with a flexible hinge made from a one-side adhesive transparency that was 75-µm thick (Grafix Light Weight Laminating Film). We used a 63-µm thick transparency during voltage vs. angle testing to create hinges with lower stiffness. The transparency was cut to the same width as the stiffening layers and was attached, adhesive side down, leaving no gap between the layers. Before the next step, the hinges were "broken in" by flexing them to > 90 degrees several times. Next, adhesive transfer tape (3M, 924) was placed over the joint structure in order to adhere the actuator. The tape was placed to cover the entire area under the pouch, slightly wider than the pouch width (~ 1 mm each side) and extending ~ 5 mm above and below the extent of the entire pouch height (h plus r). In the area under the notched region of

the pouch, the tape was removed from the sides by ~ 4 mm to minimize constraints by enabling the sides of the notched area of the pouch to lift off the surface during actuation to form a cylindrical section. By using the transfer tape as separate adhesive layer (rather than using a hinge with two-side adhesive) we were able to select precisely where the actuator attached to avoid over-constraining the pouch, as described above.

Attaching and filling the actuator: The actuator was adhered to the structure while empty to enable proper alignment. A needle and syringe were filled with liquid dielectric; actuators used liquid dielectrics with room temperature viscosities of either ~ 30 cSt (Envirotemp FR3, Cargill) or 5 cSt (Silicone oil, 317667, Sigma-Aldrich). The needle was bent at ~ 45 degrees to enable insertion into the fill port of the surface-mounted pouch. The pouch was overfilled slightly, then the bubbles were removed manually. The final fill amount was determined by weight according to **Table S1**, and measured with a precision balance (Ohaus Adventurer, S05015). The filling amounts were determined iteratively; for each actuator, the amount chosen allowed for the electrodes to fully zip at 90 degrees of flexion (measured by pressing on the filled pouch with an acrylic plate to simulate electrode zipping). After filling, the fill ports were sealed off using a flat tip on a soldering iron heated to 195 ^{o}C. Finally, copper tape (1/8”, Oubaka) was placed over the electrode leads and extended past the acrylic support. It was bonded to the electrodes with conductive carbon glue (Conductive Carbon Glue 16050, PELCO).

Torque vs. angle tests

SES joints used a 3-mm acrylic stiffening layer to provide rigidity during characterization. Torque vs. angle measurements were made using a custom test setup (**Figure S1A**). The top of the joint was mounted to an adjustable acrylic piece to accommodate different actuator geometries; this acrylic piece fixed the upper portion of the actuator. The stand was designed such that during actuation, the bottom of the SES joint contacted a 500-g load cell (Robotshop RB-SEE-198) via a measurement interface (**Figure S1B-D**) to measure force output. A Wheatstone bridge (Phidgets PhidgetBridge, 1046_0B) measured the signal from the load cell using a custom python script (Python 3.7). To measure force at different angles, the load cell was mounted on an adjustable slider that could be secured at various angles. The load cell was positioned parallel the to the bottom of the actuator at a given angle, and a small acrylic load cell connector (**Figure S1B-D**) ensured contact between the actuator and load cell at a distance of 1.5 cm (for 2x4x1 and 4x4x1 actuators) or 2 cm (for 4x4x1.5 and 4x10x1.5 actuators) from the actuator hinge. Two 0-80 screws acted as guides during actuation to provide alignment and limit lateral motion of the joint relative to the load

cell. During actuation the guide bolts were kept loose to account for any misalignment in the setup. The actuator was activated using a 0.25 Hz signal with 0.25 s rise and fall times and an amplitude of 9 kV, and the polarity of the applied voltage was reversed every actuation cycle (**Figure S1E**). The peak force was measured for each actuation cycle and averaged over four cycles. This value was converted to a torque by multiplying by 0.015 m or 0.02 m (the lever length, **Figure S1B**). Specific torque measurements normalized the maximum measured torque output of an actuator to the mass of the actuator, which was measured using a precision balance (Ohaus Adventurer, S05015).

The load cell used to measure torque was calibrated at each tested angle to read zero when not in contact with the actuator, and the reported torque was the maximum torque measured by the load cell, averaged across four cycles (**Figure S1F**). When the actuator is attached to the load cell, but not powered, the restoring force of the hinge causes a counter-torque and a negative reading on the load cell (**Figure S1F**). Since these torques are necessary to cause the hinge to bend, they were not added to the reported torque in order to provide a fair comparison.

Angle vs. voltage tests

For these tests, actuators were attached to a stiffening layer made from 1.5-mm-thick acrylic to reduce the effect of the weight of the stiffening layer on testing. Angular output of the SES joints was tested using a custom acrylic setup with the joints oriented horizontally, as shown in **Figure S2A**. The output angle was measured indirectly using a laser displacement sensor (Keyence, LK-H057). The laser measured the distance to a target over the rotating portion of the actuator; this distance was converted to an angle using the known geometry of the testing stand **Figure S2B** with the following equation:

$$\theta = \arctan\left(\frac{a - x \cdot \cos(\phi)}{x \cdot \sin(\phi) - b}\right) \tag{S1}$$

where a is the vertical distance between the output of the laser and the hinge, b is the horizontal distance, x is the distance from the laser output to the target surface on the joint, and θ is the hinge angle relative to horizontal. Actuators were activated using the ramped square signal used for torque vs. angle measurements (**Figure S1E**), with amplitudes ranging from 1 kV to 9 kV.

Measuring the permittivity of L0WS thermoplastic film

Carbon ink (CI-2051, Engineered Material Systems Inc.) was screen-printed onto either side of a sheet of L0WS film to create overlapping circular electrodes with a diameter of 3 cm. Nine of

these samples were prepared, each forming a discrete capacitor with L0WS as the dielectric. The thickness of the L0WS film for each sample was measured using a micrometer (Mitutoyo 293-340-30 Digital Micrometer) and found to be 20 µm, confirming the value specified by the manufacturer. The capacitance of each sample was measured using an inductance-capacitance-resistance (LCR) meter (BK Precision 886) set at a measurement frequency of 100 Hz at 1 V_{rms}. With the above information, we calculated the dielectric constant for each sample using the basic equation for the capacitance of a parallel plate capacitor:

$$C = \varepsilon_r \varepsilon_0 \frac{A}{d} , \tag{S2}$$

where ε_r is the relative permittivity of the dielectric, $\varepsilon_0 = 8.85 \, x \, 10^{-12}$ F/m is the permittivity of free space, *A* is the area of an electrode, and *d* is the thickness of the dielectric. Solving **Equation S2** for the relative permittivity of the dielectric yields

$$\varepsilon_r = \frac{Cd}{A\varepsilon_0} . \tag{S3}$$

The average relative permittivity for the nine samples was 3.15.

Repeatability measurements for SES joint

SES joints exhibit repeatable actuation for many cycles. **Figure S3** shows the angular output of a 2x4x1 cm BOPP joint across 2000 cycles, when activated with a modified sine wave signal with frequency 1 Hz and amplitude 8 kV (**Fig 5A**). The joint demonstrated less than a 3% change in angular output between the first 25 and last 25 cycles and lasted about 2800 cycles until failure. The joint failed electrically through the heat seal, caused by gradual damage from repeated electrical discharges through the air, emanating from the leads of the electrodes. In this study, no effort was made to prevent these discharges; we expect that suppressing these discharges through the air would improve lifetime by more than an order of magnitude.

Estimating bending stiffness of the hinge

To estimate the rotational stiffness of the hinge, we model the transparency that connects the two rigid supports as an elastic plate of length L_h, thickness t_h, width w_h (**Figure S4A**). The flexural stiffness of the plate is $D = Et_h^3/12(1-\nu^2)$, where E is the Young's modulus and ν is the Poisson's ratio of the transparency. When the hinge rotates, we assume that the transparency deforms into a cylinder section of radius R and central angle θ. The energy stored in the transparency for this deformation can be calculated as

$$U_b = \frac{1}{2}\frac{w_h D_h}{L_h}\theta^2. \quad \text{(S4)}$$

Consequently, the hinge can be treated as a rotational spring with spring constant $k_b = w_h D_h / L_h$.

For PET, typical material properties are $E \approx 2$ GPa, $\nu \approx 0.4$.[61] Therefore, for a 75-µm-thick transparency, the flexural stiffness is $D = 8.4 \cdot 10^{-5}$ N·m. In the actuators, the length of the section of the transparency that bends is $L_h \approx 1$ mm. This length isn't expressly built into the hinge, as the rigid supports have no gap in the hinge area; however, it is observed that the hinge delaminates along a length of ~ 1 mm during actuation. For an actuator with w = 4 cm, the width of the transparency was w_h = 5 cm, so we estimate a bending stiffness k_b = 4.2 mN·m/rad. For an actuator with w = 10 cm, the width of the transparency was w_h = 11 cm, so we estimate a bending stiffness k_b = 9.2 mN·m/rad.

Frequency response and impulse tests

Bandwidth and impulse response were measured using the angle vs. voltage test setup (**Figure S2A**). SES joints used 1.5-mm-thick acrylic to reduce the impact of the inertia of the stiffening layer during testing. Due to the highly nonlinear response of the joints, discrete testing frequencies were used for bandwidth tests, rather than a chirp signal, to avoid unintentional mapping of harmonics to other frequencies. Matlab (version 2019a) was used to implement a Discrete Fourier Transform (DFT) for frequency domain analysis. A modified sine wave (**Figure 5A**) was used as the actuation signal for frequency tests. Laser displacement data for frequency tests was recorded at a rate > 50x actuation frequency. Impulse tests used a reversing polarity square wave with amplitude 9 kV.

Addition of an elastic band to modify actuator response: During frequency testing, test 5 added an elastic band (5/16" light force 2.5 oz. orthodontic band, Prairie Horse Supply) on the back of the SES joint (**Figure S7**). The elastic band was pre-strained onto pegs spanning the back of the hinge. Since the band only experienced minor additional strain during flexion of the joint, it provides an approximately constant restoring force to the hinge during operation. In contrast, the transparency hinge provides a progressive restoring force that increases as the hinge angle increases (due to the increased bending of the transparency film).

Calculations of power output

Power output was measured for a 2x4x1 cm L0WS joint with 5 cSt fluid using a 20-g external load, 3.5 cm from the hinge. The angle vs. voltage measurement stand was used (**Figure S2A**) and

the joints were driven by a square wave voltage signal (**Figure S9A**) at 9 kV. The laser displacement sensor (Keyence, LK-H057) recorded at 5 kHz. Angular displacement was measured during flexion (**Figure S9B-C**), and derivatives were calculated to determine angular velocity ω (**Figure S9D**) and angular acceleration α (**Figure S9E**). Displacement data was filtered using a Savitzky-Golay filter. Power output was calculated using the simplified model in **Figure S8**. First, we calculated the torque exerted by the joint as the sum of the torque accelerating the brass disc weight (τ_{w}) and the torque required to overcome gravity (τ_{g}):

$$\tau_{\mathrm{total}} = \tau_{\mathrm{w}} + \tau_{\mathrm{g}} \quad \text{(S3)}$$

$$\tau_{\mathrm{total}} = I\alpha + Mg\sin(\theta)R_1 \quad \text{(S4)}$$

where I is the moment of inertia of the weight for rotation around the hinge, α is the angular acceleration of joint, θ is the angle of the hinge in radians, M is the mass of the weight (20 g), g is the acceleration due to gravity (9.8 m/s^2), and R_1 is the distance from the hinge (3.5 cm) to the center of the weight. Using the parallel axis theorem,

$$I = \frac{MR_2^2}{4} + MR_1^2 \quad \text{(S5)}$$

where R_2 is the radius of the weight (1.3 cm). Power output can be calculated as

$$P = \omega\tau_{\mathrm{total}} \quad \text{(S6)}$$

where ω is the angular velocity of the joint. Then the power output becomes

$$P = \omega\left[\left(\frac{1}{4}MR_2^2 + MR_1^2\right)\alpha + Mg\sin(\theta)R_1\right]. \quad \text{(S7)}$$

In these calculations, we ignore the moment of inertia of the stiffening layer and assume that the brass weight is in plane with the hinge surface. To calculate the specific power, the power output of the joint is divided by the mass of the filled actuator (1.36 g), rather than the mass of the entire joint. The reasoning behind this choice is that 1) in applications, the support structure would likely be part of the mechanical structure of the robot, therefore does not contribute additional mass to the robotic system, and 2) the mass of the joint is largely arbitrary based on the choice of materials and dimensions of the stiffening layer during testing.

Power consumption

Tests of power consumption used a lightweight servo motor (Pololu #1053, Sub-Micro Servo 3.7g). The SES joint was a lightweight design that used a 2x4x1 L0WS actuator attached to a 3 mm balsa wood stiffening layer laser cut into shape (Trotec Speedy 360 Flexx, 75W CO2). The servo

motor was powered using a 5V benchtop DC power supply (Keysight U8002A) and controlled using the PWM signal from a microcontroller (Elegoo Mega 2560) and the "Servo" control library in Arduino (version 1.8.1).

Power consumption for the servo motor was measured using the circuit shown in **Figure S10**. An NI DAQ (Model USB-6212 BNC) monitored the voltage across the motor (V_S) and a measuring resistor (V_R) using a custom Matlab program (version 2019b). A 206 mΩ measuring resistor was chosen to limit the voltage drop to < 100 mV, giving a high signal-to-noise ratio but ensuring nearly the full 5 V across the motor. Current is calculated as $I = V_R/206$ mΩ, and instantaneous power is calculated as $P = IV_S$. Current and voltage were sampled at 200 kHz. The resulting signals were processed using a rolling average and Savitzky-Golay filters to smooth current and power data.

Power consumption for the SES joint was measured using the circuit shown in **Figure S11**. A Trek high voltage power supply (Model 50/12) provided a voltage across the electrodes of the actuator. On the ground side of the actuator, a current meter (uCurrent Gold rev. 2) measured the current provided by the power supply. The voltage output to the actuator, V_A, was measured using the voltage monitor from the Trek. The current meter was set to the 1mV/μA range to produce a signal output voltage, V_I. Both voltages were read out using an NI DAQ (USB-6212 BNC) sampling at 10 kHz per channel. A 1 MΩ resistor in series on the ground side of the actuator protected the current meter from a surge in the event of electrical failure of the actuator. A 1 mF electrolytic capacitor filtered the voltage noise from the output of the current meter.

Antagonist arrangement of SES joints for bidirectional actuation

Fabrication: To enable actuation in both directions with minimal constraint, the bidirectional joint required a modified fabrication process and materials (**Figure 7B**). Pouches were fabricated from BOPP film using the 2x4x1 cm geometry. After sealing, these were then filled with liquid dielectric and set aside without electrodes. To make the support structure, a two-side adhesive film (Grafix Double Tack Mounting Film) was used as the elastic hinge material. Flexible stiffening layers of transparency film with a thickness of 125 μm (Grafix Heavy Weight Laminating Film) were attached to either side of the hinge, leaving a 2-mm gap between top and bottom portions to create the hinge length.

Mounting actuators: To mount the actuators, two-side adhesive tape (3M, 924) was placed on one side of the support structure. In this case, a 2.5-mm gap was left in the tape on either side of the hinge to ensure that the actuator didn't adhere to that region when mounting. The bidirectional actuator used an adhesive transfer tape with a conductive scrim (3M xyz-axis conductive tape, 9719) as the electrode for the stiffening layer side. This electrode was cut into shape. The electrode

was placed onto the stiffening layer in the appropriate region, with a lead extending upward in the middle. The pouch was attached to the electrode and surrounding adhesive on the upper portion of the stiffening layer (above the hinge). To add slack into the structure, the hinge was biased away from the actuator side by 45 degrees before attaching the actuator to the lower portion of the stiffening layer (below the hinge). This process was repeated for the actuator on the other side of the structure with the electrode leads being electrically connected in the center. Finally, the exterior electrodes were fabricated from ionically conducting hydrogel[85] that was formed into an electrode using the process described by Kellaris et al.[57] These electrodes were placed on the exterior of the pouch, with leads extending upward on opposite sides (**Figure 7B**).

Driving the actuators: The electrical driving scheme for the bidirectional SES joint can be seen in **Figure S12**. The input voltage to the joints was generated by a Trek HV amplifier (Model 50/12), which was set to a DC voltage of 8 kV. Optocouplers (OC100HG, Voltage Multipliers Inc.) were used to connect each electrode to either the HV or ground. The LEDs of each optocoupler were operated using the circuit presented by PetaPicoVoltron.[86] This driving scheme was designed to reverse the polarity of the voltage applied to each actuator during subsequent actuation cycles. Reversing polarity was shown to mitigate the effects of charge retention within the BOPP-based actuators. The bidirectional SES joint was operated at a range of frequencies, up to 15 Hz, alternating between actuation of the left and right actuator.

Artificial spider limb

Fabrication: The actuators used for the artificial spider limb were built from L0WS as the dielectric shell, FR3 as the liquid dielectric, and screen-printed carbon ink as the electrodes. The dimensions of the pouches are as follows:

- Actuator 1 had dimensions of 4x10x1.5 cm
- Actuator 2 had dimensions of 4x6x1 cm
- Actuator 3 had dimensions of 2x4x1 cm

The high-voltage electrodes and leads (outward facing) of each joint were coated with a 200-μm-thick layer of silicone elastomer (Ecoflex 00-30, Smooth-On) to prevent electrical arcing between adjacent HV leads when the joints were activated independently. The L0WS dielectric film shows substantially less charge retention than the BOPP film, and so reversing polarity of the activation voltage was not required between subsequent actuation cycles. Therefore, the actuators

of the artificial spider limb shared a common ground. The acrylic supports were cut from 1.5-mm-thick acrylic. All hinges were constructed from 75-µm-thick adhesive transparencies (Grafix Light Weight Laminating Film) with no gap between the acrylic stiffening layers.

Driving the actuators: The actuators were driven independently using the three-channel power supply created previously by Mitchell et al.[46] When operating the actuators simultaneously, a pressure-sensitive resistor (SEN-09375, SparkFun) was used to modulate voltage output to demonstrate controllable, lifelike motion, and fast response to stimuli.

Three-finger gripper

Fabrication of the fingers: The actuators for the three-finger gripper were designed with a taper so that the basal joint could provide the necessary torque for gripping objects. Each finger was comprised of two actuators made from 20-µm L0WS film with pouch dimensions 3x5x1.5 cm and 2x4x1 cm (**Figure S13A**). Both actuators were made from the same film with incorporated filling ports to allow for filling each individually while mounted onto the stiffening layers. The film was sealed around the actuators in an outline to prevent oil from getting between the film layers while filling – this outline was cut away to leave a 1-cm-wide skirt after filling. Electrodes were connected so they could be simultaneously activated (**Figure S13B**). After printing the electrodes, the actuators were adhered onto a 1.5-mm acrylic stiffening layer (**Figure S13C**), which was designed with a similar taper to allow the three fingers to close to a grasp when fully activated.

Compliant end effector: The compliant end effector was a simple strip of transparency film, looped over the end of the fingers and adhered at its ends using the adhesive transfer tape. The loop created a compliant structure that could deform when contacting a surface (observable in **Movie S8**). A 0.5-mm-thick strip of elastomer (Ecoflexx 00-30) was taped over the transparency loop to provide a high-friction interface.

Design of gripper base: The fingers were attached to a base to create the three-finger gripper. The base was designed in CAD software (Solidworks 2019), exported to an .stl file, and printed on an SLA 3D printer (Form 2, Formlabs) using gray resin. The base was designed with a 15-degree taper to bias the fingers outward slightly in order to increase the size of the objects that could be gripped, while maintaining the ability to close into a full grasp when activated. The fingers were mounted to the base using two M4 plastic screws for each finger. The electrical leads of each finger were joined with copper tape (1/8”, Oubaka) such that there was one common HV connection and ground lead.

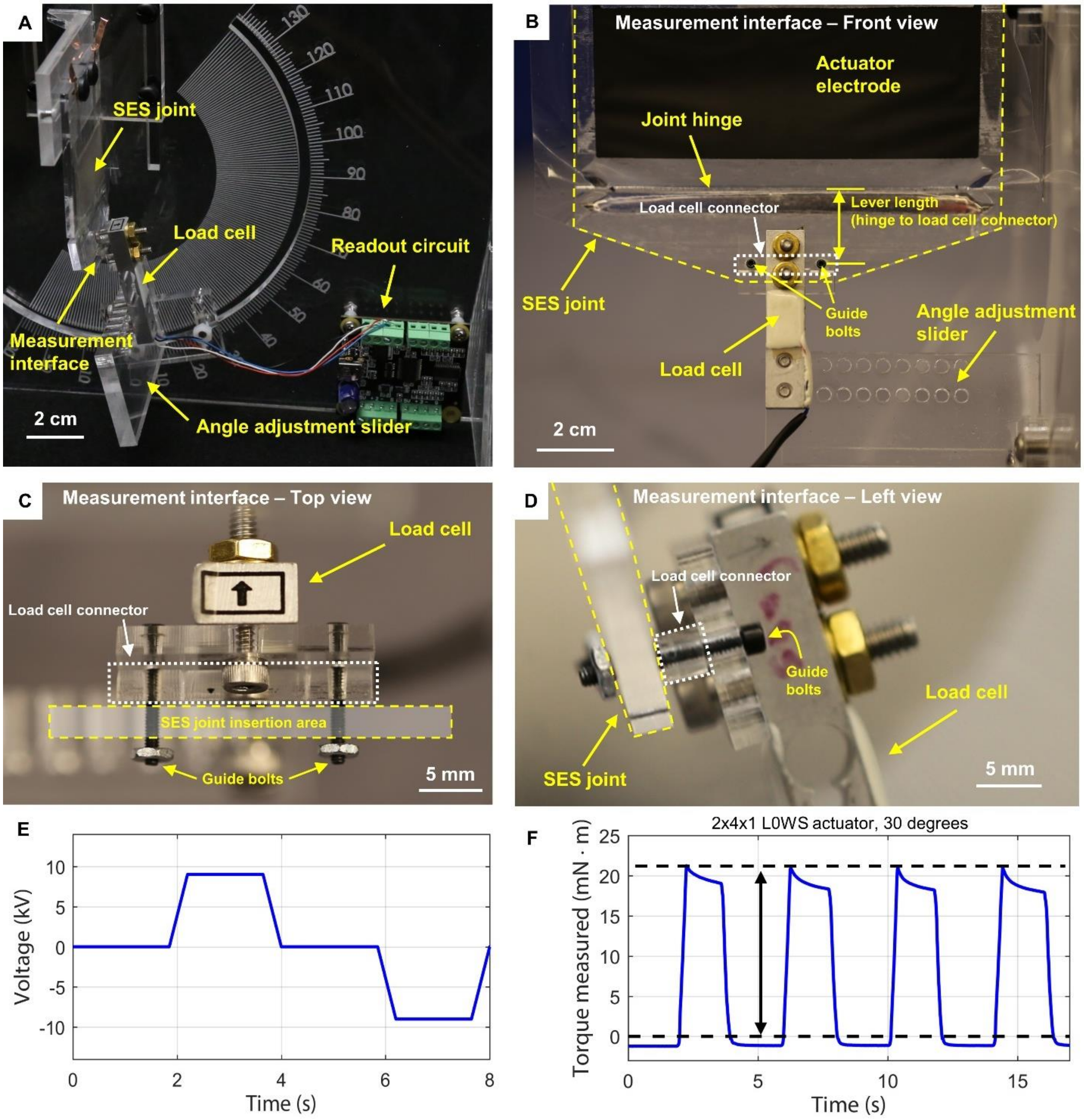

Figure S1. Torque measurements for SES joints. (A) Custom stand used for measuring torque output of the joints; a load cell on a sliding mount allows for adjustable testing angles. A 2x4x1 cm SES joint is attached. **(B)** Front view of the measurement interface (with a 4x10x1.5 cm SES joint attached). The joint was loosely connected to the load cell with two bolts for maintaining test alignment and for providing guide rails during measurements. The lever length for torque measurements is the distance between the center of the joint hinge and the center of the acrylic load cell connector held by the guide bolts. The setup is designed such that the joint only contacts the narrow load cell connector during testing. **(C)** A top view shows the measurement interface as well as where the stiffening layer of the joint connects. The guide bolts travel through two holes in the

joint stiffening layer and are loosely held in place using two hex nuts (seen at the bottom of the image). **(D)** Side view from the left of the measurement interface. The load cell connector extends to the left slightly farther than other components of the measurement interface such that the SES joint only contacts the connector during measurement. **(E)** Voltage signal profile used during torque testing. Polarity of the high voltage signal reversed every cycle (9 kV → -9 kV → 9 kV …) **(F)** Torque measurements recorded during actuation cycles for a 2x4x1 cm L0WS joint measured at 30 degrees. The reported torque was the maximum positive torque measured in a cycle, averaged across four cycles (magnitude is measured between the dashed lines). The small negative reading in each cycle is caused by the counter-torque of the hinge pulling back on the load cell as it tries to straighten out when the actuator is not powered.

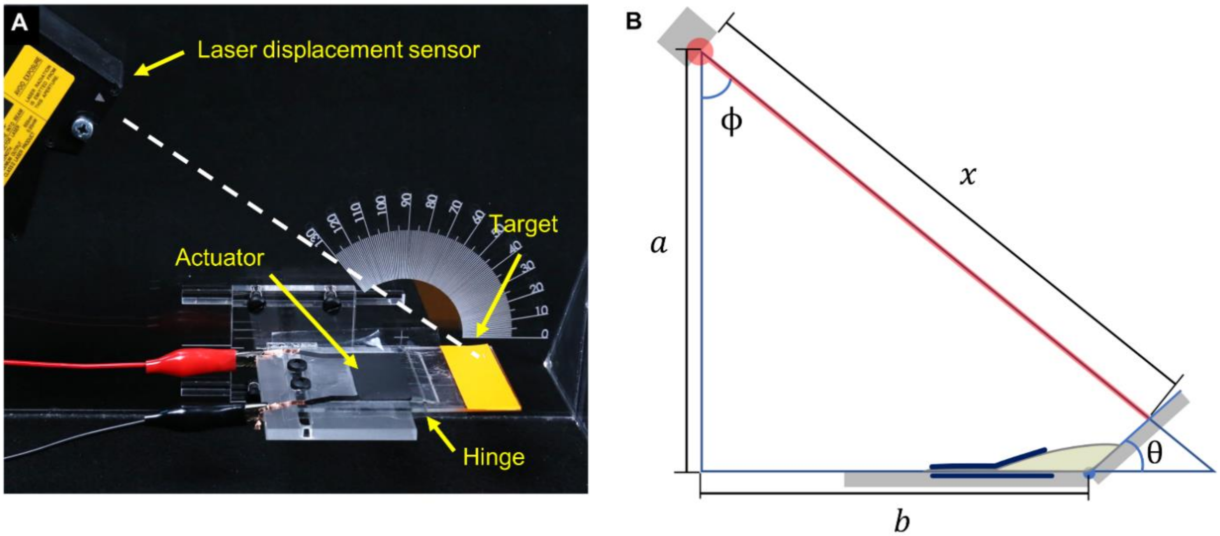

Figure S2. Measurement setup for angle vs. voltage testing of SES joints. (A) A laser displacement sensor was mounted to an acrylic stand. The distance to a target on the stiffening layer of an SES joint was measured. **(B)** The known geometry of the test stand was used to transform distance *x* (measured with the laser displacement sensor) into joint angle θ, using **Equation S1**.

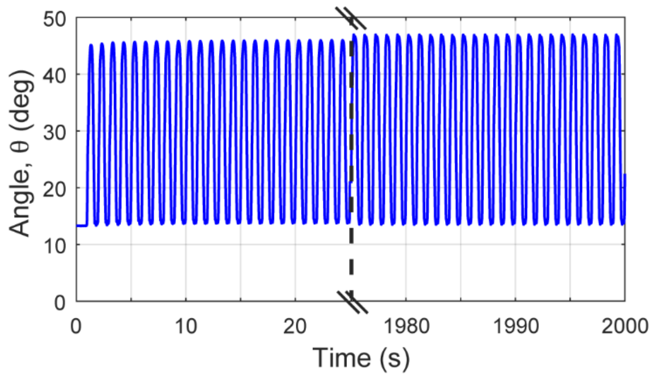

Figure S3. Testing the repeatability of acutation in SES joints. Angle versus time for a 2x4x1 cm L0WS actuator with 5 cSt liquid dielectric using a 1 Hz signal over 2000 cycles. The amplitude of displacement only changes by 3% between the first 25 cycles and the last 25 cycles, demonstrating the repeatability of actuation in these systems.

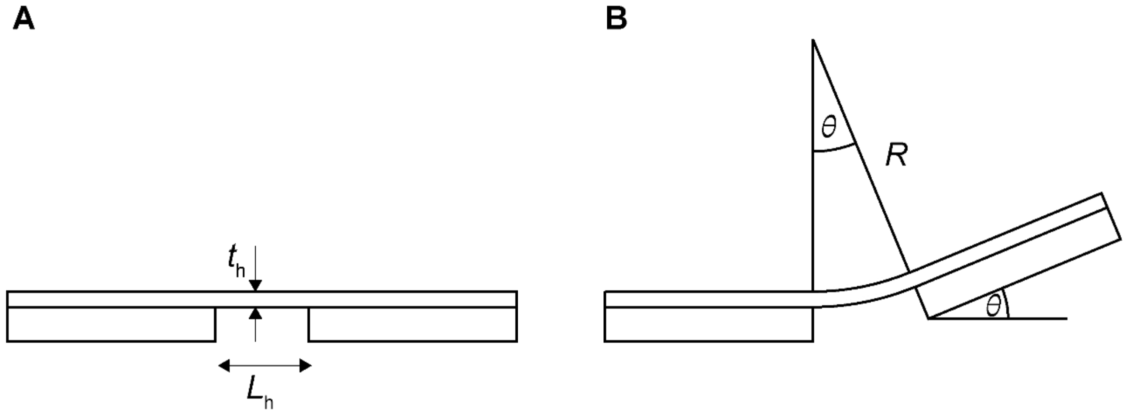

Figure S4. Estimation of the rotational stiffness of the hinge. (A) The length of the transparency that connects the two rigid supports is L_{h}, and its thickness is t_{h}. The width w_{h} of the hinge is out of plane. **(B**) When the hinge rotates by an angle θ, the transparency deforms into a cylinder section with radius R and central angle θ.

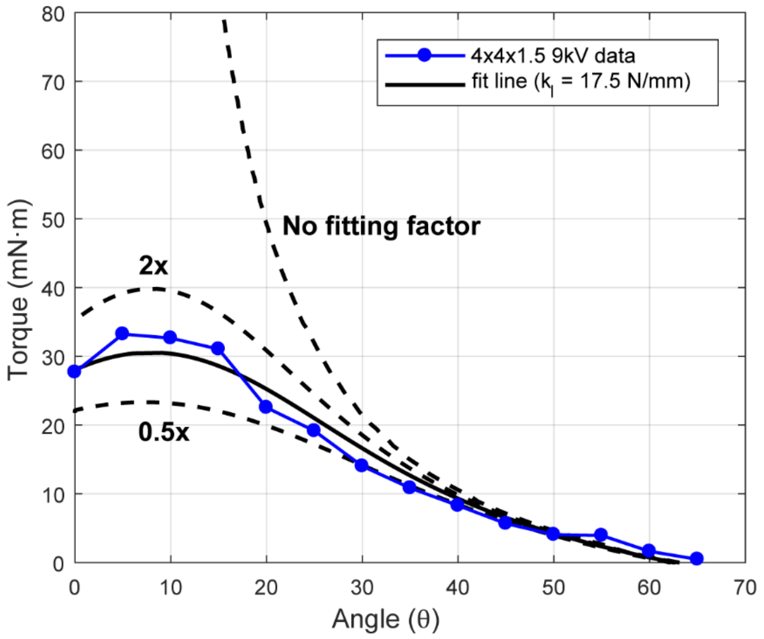

Figure S5. Model behavior for different k_l values. Plot comparing experimental and model curves for a 4x4x1.5 cm BOPP/FR3 actuator activated at 9 kV. The solid black line presents the fit for the chosen value of the fitting parameter k_l (17.5 N/mm). The fit lines for different values of k_l (0.5x and 2x the best fit value) give realistic but worse fits to the data. Without any fitting factor ($k_l \rightarrow \infty$), the model predicts unrealistic asymptotic behavior at low angles.

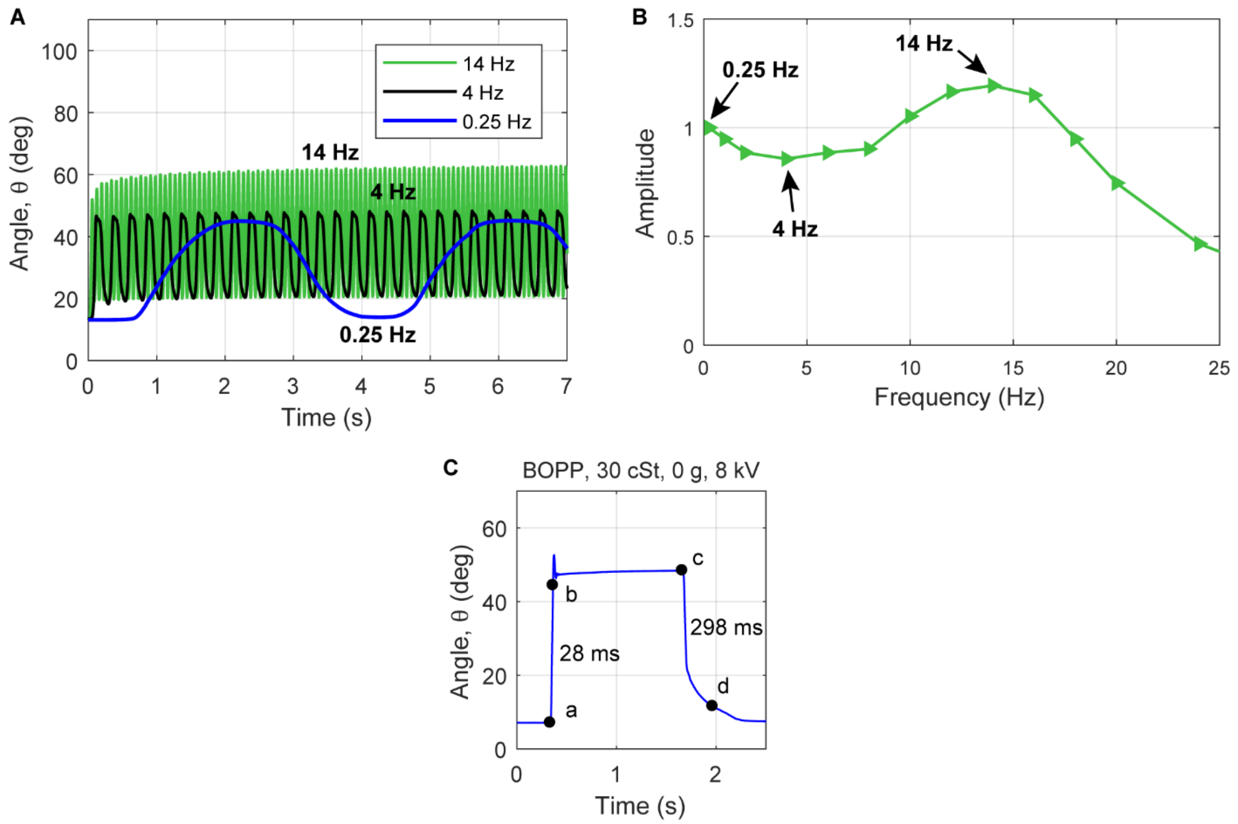

Figure S6. Dynamics of SES joint using BOPP and FR3 liquid dielectric. (A) The angle vs. time response for a 2x4x1 cm BOPP-based SES joint with 30 cSt liquid dielectric (FR3) at several frequencies when driven by the modified sine wave voltage (**Figure 5A**). Above 0.25 Hz, the minimum angle reached shifts upward, due to the viscosity of the liquid dielectric. **(B)** These characteristics lead to the actuator exhibiting characteristics of dampening below 4 Hz while still maintaining a resonance peak. **(C)** During impulse testing (driven with a square wave voltage) the joint exhibits strongly asymmetric rise and fall times – 28 ms and 298 ms respectively. We attribute this asymmetry to viscous effects during passive relaxation of the joint.

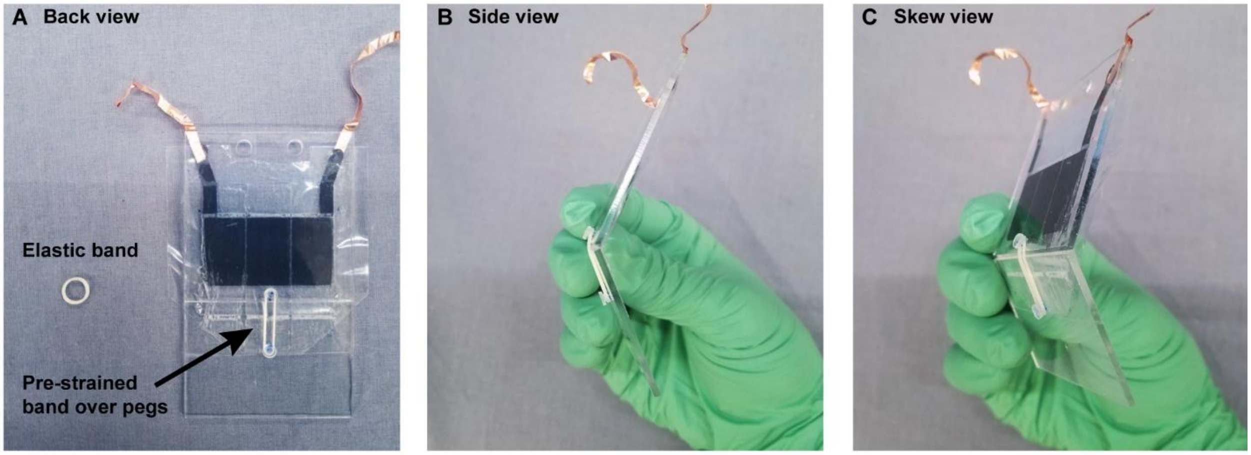

Figure S7. Adding elastic band to SES joints to modify type of restoring force. (A) An elastic band is pre-strained over pegs on the back of the joint. The band experiences only minor increased strain during flexion of the joint, and thus provides an approximately constant restoring force over the entire bending range (in contrast to the bending hinge, where restoring force increases progressively with larger bending angles). **(B)** Side view and **(C)** skew view of the deformation in the band during flexion.

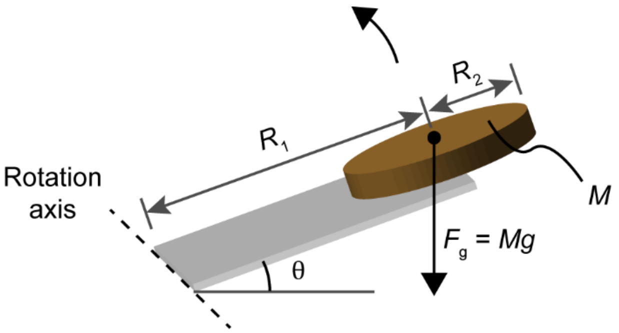

Figure S8. Model used for calculating power output of SES joints. A brass disc weight (radius $= R_2$, mass = M) is attached to the rotating portion of the joint, a distance R_1 from the hinge. Gravity (acceleration = g) acts vertically on the weight throughout actuation with force $F_g = Mg$ as the hinge rotates, changing angle θ.

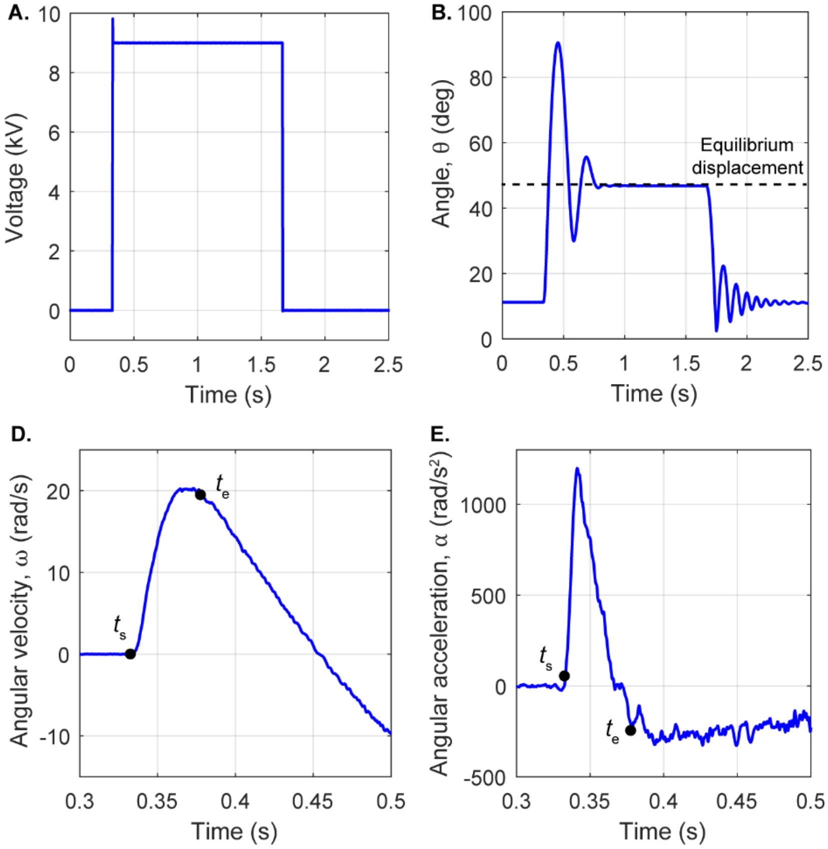

Figure S9. Analysis of specific power for SES joints. (A) A square wave voltage signal with amplitude 9 kV was used to excite the joint. **(B)** Angle vs. time response for a 2x4x1 cm L0WS-based joint using 5 cSt liquid dielectric with a 20-g load. **(C)** Zoom view of flexion angle during activation. The time to equilibrium is measured from the time of initial application of voltage (t_s) to the time of equilibrium displacement (t_e). **(D)** Angular velocity vs. time. **(E)** Angular acceleration vs. time. **(F)** Specific power during flexion for a 20-g inertial load. To calculate specific power, only the weight of the actuator (1.36 g) in the SES joint was considered.

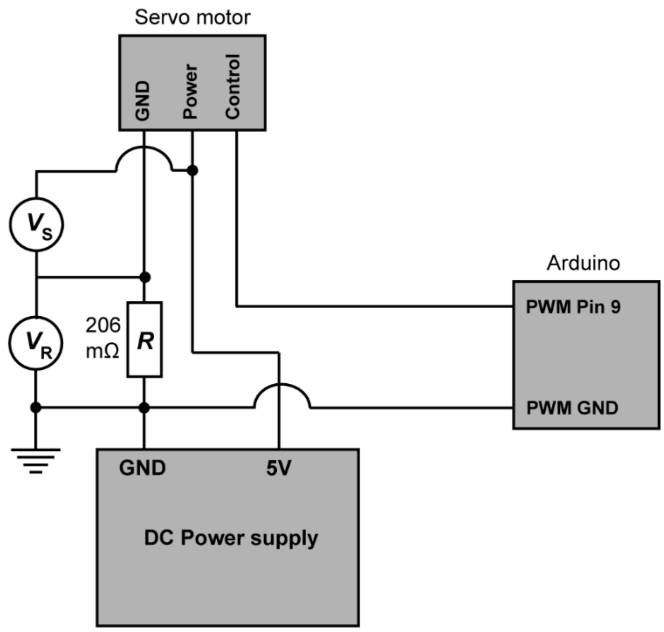

Figure S10. Circuit schematic for measuring power consumption of the servo motor.

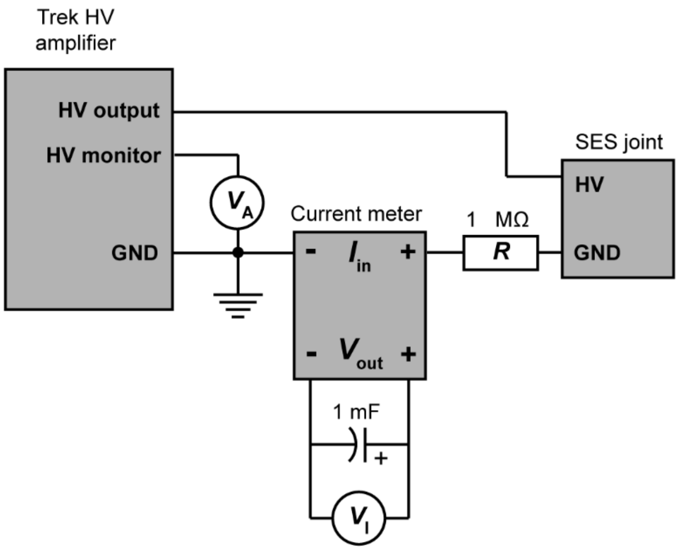

Figure S11. Circuit schematic for measuring power consumption of SES joints.

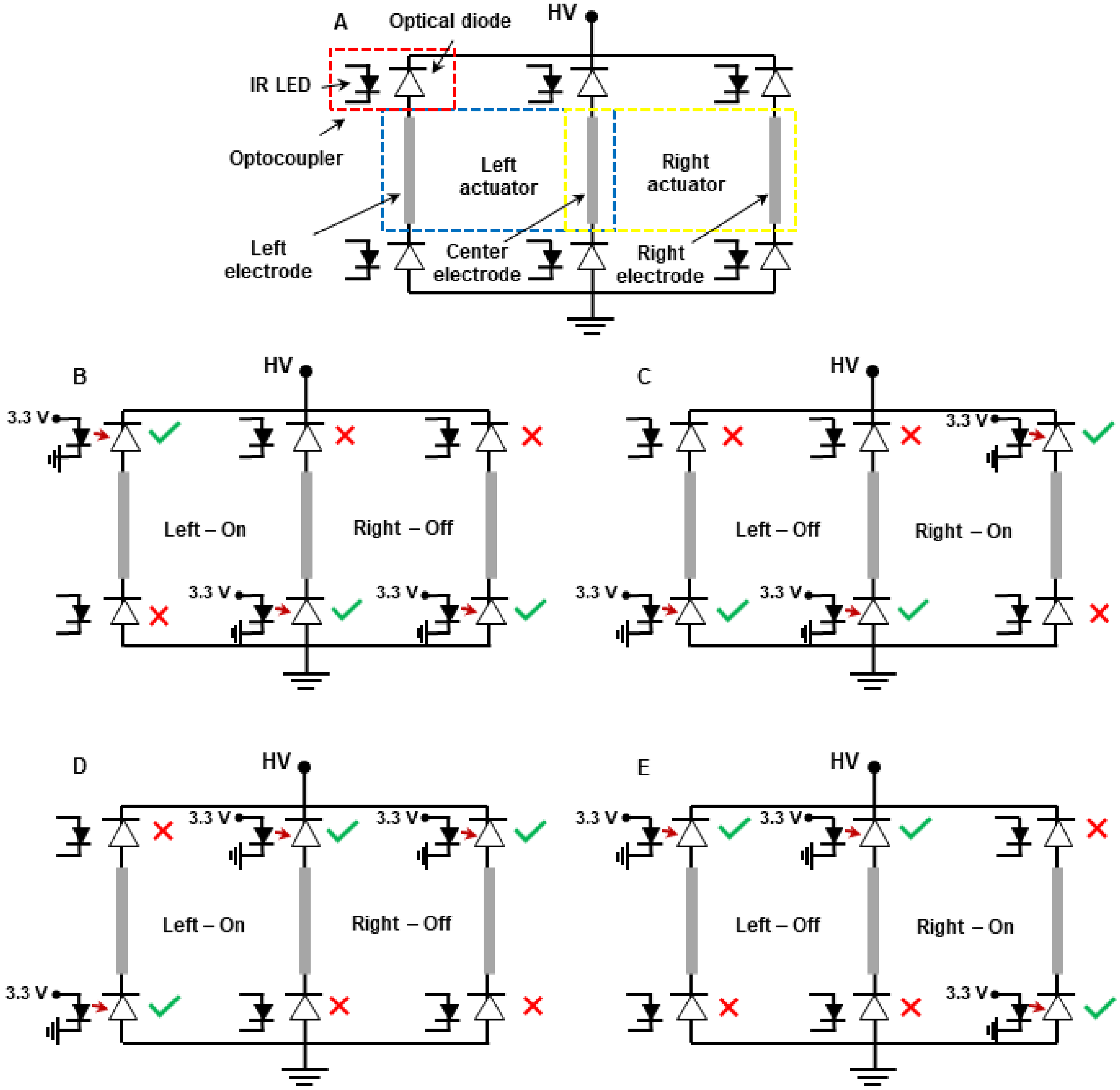

Figure S12. Electrical driving scheme for SES joint with bidirectional actuation. **(A)** The circuit utilized two optocouplers (OC100HG, Voltage Multipliers Inc.) for each electrode, one to connect the electrode to high voltage (HV) and one to connect the electrode to ground. The left and right actuators shared a central ground electrode. When the infrared (IR) light emitting diodes (LEDs) of the optocouplers were powered by 3.3 V, the optical diode conducted HV current. **(B)** The left actuator was charged by connecting the left electrode to HV, while the center and right electrode were connected to ground. In this state, the right actuator was discharged. **(C)** The right actuator was charged by connecting the right electrode to HV, while the center and right electrode were connected to ground. In this state, the left actuator was discharged. **(D)** The voltage applied to

the left actuator in the subsequent actuation cycle is the opposite polarity as in (B). The left electrode was connected to ground, while the center and right electrode were connected to HV. **(E)** The voltage applied to the right actuator in the subsequent actuation cycle is the opposite polarity as in (C). The right electrode was connected to ground, while the center and left electrode were connected to HV. For periodic bidirectional actuation, steps (B)-(E) were repeated.

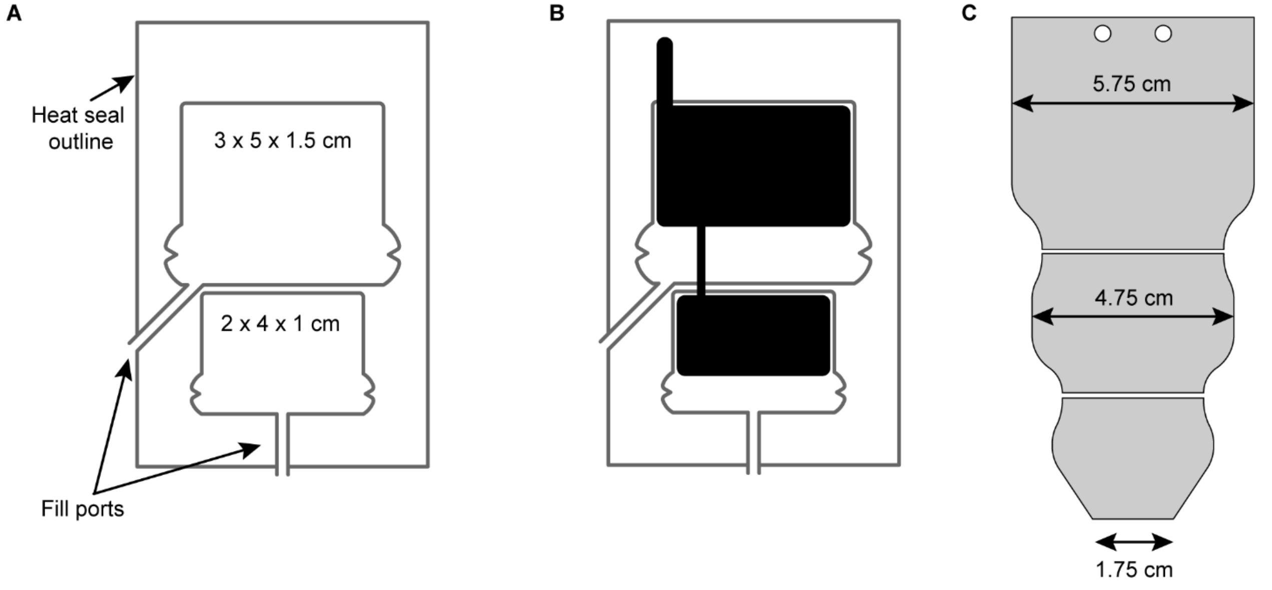

Figure S13. Schematics of individual fingers for three-finger gripper. (A) The heat seal pattern for the gripper pouches included two pouches: The first joint used a 3x5x1.5 cm pouch while the second joint used a 2x4x1 cm pouch. Both were made from the same L0WS film. Fill ports were included for each pouch such that they could be filled after mounting. A heat seal outlined the entire pattern to avoid getting oil between the films during filling. **(B)** Electrode pattern deposited on each side of the film. **(C)** Acrylic stiffening layer segments used a tapering design to allow the fingers to create a closed grasp when fully flexed.

**Actuator dimensions** ***h*** **x** ***w*** **x** ***r*** **(cm)**	**Liquid dielectric fill (grams)**	**Liquid dielectric fill (mL)**	**Mass of actuator (grams)**
2 x 4 x 1	1.15	1.2	1.28 (1.36 for L0WS)
3 x 5 x 1.5	3.30	3.5	-
4 x 4 x 1	1.25	1.3	1.42
4 x 4 x 1.5	2.70	2.9	2.90
4 x 6 x 1	1.88	2.0	2.13
4 x 10 x 1.5	6.75	7.2	7.17

Table S1. Liquid dielectric fill and actuator mass for different types of SES joints used in the paper. Liquid dielectric fill: The amount of liquid dielectric was determined by mass to account for any liquid lost during the filling process. The calculated volume was based on a measured density of 0.94 g/mL for FR3 liquid dielectric. The same mass of liquid dielectric was used for the 5 cSt silicone oil (reported density 0.913 g/mL) for simplicity. Mass of actuator: The mass of the actuators was measured using BOPP film. The actuator mass in parentheses is for a 2x4x1 L0WS actuator. The L0WS actuator was slightly heavier as L0WS film is thicker and polyester has a higher density than polypropylene.

Captions for Supplementary Movies

Movie S1. Demonstration of SES joint operation. An SES joint demonstrates controllable flexion when activated at 8 kV using a slow (0.25 Hz) ramped voltage signal and rapid flexion when using a fast (4 Hz) square wave voltage signal.

Movie S2. Backdrivability of SES joints. SES joints are backdrivable, which allows them to adjust their angular output based on unexpected interactions with external objects.

Movie S3. Demonstration of a four-legged robot. SES joints can be combined to create different types of robotic structures such as a four-legged robot that features fast and controllable actuation.

Movie S4. A jumping robot powered by SES joints. SES joints can be employed in a lightweight device with high enough power density to jump 5.5 cm into the air when activated at 8 kV with a square wave voltage signal.

Movie S5. Impulse response of an SES joint using FR3 liquid dielectric. SES joints made with FR3 liquid dielectric (viscosity ~ 30 cSt) display highly asymmetric rise and fall times. A prominent shoulder is observed in the relaxation of the actuator around 20 degrees, likely due to the viscosity of the liquid.

Movie S6. Antagonist arrangement of SES joints for bidirectional actuation. A bidirectional SES joint is actuated in both directions using frequencies ranging from 2 Hz to 15 Hz.

Movie S7. Series arrangement of SES joints for a bioinspired artificial limb. An artificial limb is constructed from three electrically independent SES joints in series. The actuators demonstrate independent control with a pre-programmed signal, followed by controllable and lifelike response using a pressure-responsive button.

Movie S8. A three-finger gripper based on series arrangements of SES joints. A three-finger gripper demonstrates horizontal stability by “picking” a strawberry. Vertically, it can grasp objects with a variety of sizes and weights without modifying the voltage signal.
